# A Randomized Controlled Trial to Test the Effectiveness of an Immersive 3D Video Game for Anxiety Prevention among Adolescents

**DOI:** 10.1371/journal.pone.0147763

**Published:** 2016-01-27

**Authors:** Hanneke Scholten, Monique Malmberg, Adam Lobel, Rutger C. M. E. Engels, Isabela Granic

**Affiliations:** 1 Department of Developmental Psychopathology, Behavioural Science Institute, Radboud University, Nijmegen, The Netherlands; 2 Trimbos Institute (Netherlands Institute of Mental Health and Addiction), Utrecht, The Netherlands; University Hospital of Bellvitge-IDIBELL; CIBER Fisiopatología Obesidad y Nutrición (CIBERObn), Instituto Salud Carlos III; Department of Clinical Sciences, School of Medicine, University of Barcelona, Spain, SPAIN

## Abstract

Adolescent anxiety is debilitating, the most frequently diagnosed adolescent mental health problem, and leads to substantial long-term problems. A randomized controlled trial (*n* = 138) was conducted to test the effectiveness of a biofeedback video game (*Dojo*) for adolescents with elevated levels of anxiety. Adolescents (11–15 years old) were randomly assigned to play *Dojo* or a control game (*Rayman 2*: *The Great Escape*). Initial screening for anxiety was done on 1,347 adolescents in five high schools; only adolescents who scored above the “at-risk” cut-off on the Spence Children Anxiety Survey were eligible. Adolescents’ anxiety levels were assessed at pre-test, post-test, and at three month follow-up to examine the extent to which playing *Dojo* decreased adolescents’ anxiety. The present study revealed equal improvements in anxiety symptoms in both conditions at follow-up and no differences between *Dojo* and the closely matched control game condition. Latent growth curve models did reveal a steeper decrease of personalized anxiety symptoms (not of total anxiety symptoms) in the *Dojo* condition compared to the control condition. Moderation analyses did not show any differences in outcomes between boys and girls nor did age differentiate outcomes. The present results are of importance for prevention science, as this was the first full-scale randomized controlled trial testing indicated prevention effects of a video game aimed at reducing anxiety. Future research should carefully consider the choice of control condition and outcome measurements, address the potentially high impact of participants’ expectations, and take critical design issues into consideration, such as individual- versus group-based intervention and contamination issues.

## Introduction

Anxiety disorders are one of the most common adolescent psychiatric disorders, affecting up to 17% of adolescents in Western countries [1,2]. Adolescent anxiety is associated with school dropout, lower school grades, suicidality, early substance use, teen pregnancies, and behavioral problems, such as attention deficit hyperactivity disorder and conduct disorder [3–7]. Moreover, adolescent anxiety tends to persist into adulthood and is related to other health problems, such as substance misuse and depression [7,8]. Despite the number of individuals suffering from anxiety and the accompanying personal, economic, and social costs, the focus in mental health has been primarily on treatment rather than prevention [9]. There are several reasons why the prevention of anxiety is pivotal [10]. First, parents and teachers are often unaware of adolescents’ anxiety problems [11] and even if awareness of the anxiety problems does exist, parents and teachers tend to downplay its importance [9]. Second, when adolescents are referred for treatment, the anxiety disorder is often well established, and the majority of adverse effects on peer relationships and school performance have already occurred and are hard to reverse [9]. Identifying effective prevention strategies for adolescent anxiety is therefore crucial.

A new and upcoming prevention strategy for adolescent mental health is the use of video games. Video games are immersive and motivate a wide range of behaviors in adolescents, with 97% of youth (both boys and girls) playing them in their leisure time [12]. Although the vast majority of psychological research on video games has focused on the negative impact, (e.g., addiction [13]; aggression [14]), there is a growing body of evidence for the potential benefits (i.e., cognitive, social, motivational, and emotional) these games may confer upon users, especially adolescents [15–21]. A recent review on the small but significant empirical work on the benefits of video games showed the enormous potential for video games in mental health contexts that can teach youth new forms of emotional strategies, behavior, and thinking [22].

Importantly, video games could address barriers that are frequently encountered in conventional prevention strategies. Specifically, Cognitive Behavioral Therapy (CBT)—the most widely used effective prevention strategy for anxiety problems—suffers from several limitations. Most important are: (1) **Engagement**: CBT largely relies on imparting psychoeducational information. Adolescents, especially those who do not recognize that they have a mental health problem, often find this boring. Motivating adolescents to engage is one of the most challenging tasks clinicians face [23]. An engaging video game could address the boredom experienced in CBT. (2) **Practice**: Even though CBT does a good job at imparting new, useful knowledge, there is a large gap between what an adolescent *knows* and what an adolescent actually *does*. This problem has long been recognized and therefore many CBT programs include role-playing exercises and homework assignments [24]. However, these exercises are mostly de-contextualized and rarely involve skill training in authentic emotional experiences. In a video game, on the other hand, adolescents are not just exposed to knowledge, but they practice skills over and over again as they are increasingly emotionally challenged; as a result, effective cognitive and emotional skills can become automatized. (3) **Need**: Many adolescents have difficulties accessing prevention programs because they live in hard-to-reach rural locations or are physically or psychologically unable to commute [25,26]. Lengthy waiting lists are also a problem [27]. Video games can be played wherever and whenever it is preferred and no waiting lists are necessary [28]. (4) **Cost**: Cost-effectiveness is often a barrier in delivering prevention programs. Many adolescents and families cannot afford participation in prevention programs and schools worldwide are facing cuts in funding [25,27]. Video games are simply far cheaper to deliver than CBT prevention programs [29]. Overall, the use of video games holds great promise for a radically new approach to delivering prevention programs for adolescents with anxiety and overcoming barriers inherent in CBT approaches.

### *Dojo*: Targets of intervention

The present randomized controlled trial (RCT) is one of the first to test this novel prevention approach by evaluating *Dojo* (developed by GameDesk; http://gamedesk.org/project/dojo/), a video game specifically designed to reduce anxiety in adolescents. *Dojo* is an emotion management video game and incorporates two evidence-based strategies for reducing anxiety symptoms; emotion regulation training and heart rate variability (HRV) biofeedback. Emotion regulation training refers to a diverse set of processes that influence the occurrence, duration, intensity, and expression of emotion [30]. Examples include progressive muscle relaxation, guided imagery, deep breathing, and positive self-talk. These techniques are well validated in adolescent anxiety treatment and are therefore commonly used in anxiety prevention programs [31–34].

Heart rate variability (HRV) is an electrocardiographic index of autonomic control of the heart and reflects changes in consecutive heart beat intervals [35]. Adolescents with anxiety disorders generally have short intervals between consecutive heart beats (i.e., low HRV). Low HRV is a hallmark of unhealthy stress recovery and is a marker of poor health [36,37]. HRV biofeedback is a technique that teaches adolescents how to alter physiological activity in order to improve health, performance, and learning [38]. It focuses on HRV enhancement by training adolescents to gain awareness of their bodily arousal, to reduce their physiological arousal, and to become more flexible in their physiological response [39,40]. The aim of training HRV through biofeedback in a video game context is to provide a model for self-regulation and an opportunity to practice these skills so that they can be automatically applied outside the game context [20,40].

*Dojo* trains emotion regulation strategies (i.e., deep-breathing techniques, progressive muscle relaxation, positive thinking, and guided imagery) with instructional videos and by then engaging players in immersive and emotion evoking puzzles that challenge players to use these newly acquired strategies. To encourage players to effectively regulate their emotions, each challenge becomes increasingly difficult if the player’s heart rate increases. Thus, based on clinical research that shows that the combination of HRV biofeedback with emotion regulation training is effective in reducing anxiety—more so than either strategy alone [41–43]–these techniques are the foundations of the game design in *Dojo*.

### Methodological considerations

Despite this promise of using video games as mental health interventions, caution is also warranted. The most important reason is that systematic, randomized controlled trials are sparse on the effectiveness of video games for the prevention of mental health concerns in general and for anxiety in particular [22,44]. Even in the medical and educational fields, where “serious” or “applied” games have been more readily accepted as training tools, very few of those games have ever been scientifically evaluated [45–47]. As a result, it is still unclear how much more effective these games are in changing health-related, educational, and behavioral outcomes than conventional approaches. To enable these fields to advance, outcome evaluations of applied video games need to employ state-of-the-art designs (e.g., RCTs) with appropriate rigor that are in line with CONSORT guidelines and with adequately powered samples [22,44–48]. Moreover, reliable and valid measures of outcomes and moderators and follow-up durations that are sufficiently long to warrant confidence in the claims about video games and the scientific evidence for their effectiveness are essential [44,47].

Another crucial factor to adequately assess potential benefits of video games is choosing an appropriate control group [45,46]. Active control groups are more scientifically rigorous than wait lists or no-attention control groups [49–51]. They insure that attention, or additional placebo mechanisms such as beliefs, behavioral activation, and expectations, do not account for improvements in the experimental condition [49–51]. Yet, active controls are only superior to “no-contact” controls when participants in the active control condition have the same expectations of improvement as participants in the experimental condition. Only then can differential improvements be attributed to the strength of the intervention. In sum, the most convincing evidence for the effectiveness of a video game as an intervention tool will come from studies using active control participants who receive a similar intervention, but that does not specifically target anxiety.

The current study aimed to address all limitations inherent to the vast majority of past evaluations of the effectiveness of video games. In accordance with the intention-to-treat principle, we conducted a sufficiently powered RCT. We included reliable and valid measures of outcomes and carried out a three month follow-up assessment. We selected *Rayman 2*: *The Great Escape* (hereafter shortened to *Rayman*) as the active control game. In comparison to the experimental game *Dojo*, *Rayman* was not explicitly designed to incorporate training components that target anxiety. Importantly, before the start of the intervention and before adolescents knew to what condition they were assigned, expectations were equalized, measured and controlled for. If *Rayman* is an adequate placebo control for the experimental game *Dojo*, participants should expect the same levels of improvement for both games before the start of the intervention.

### Design and Hypotheses

The present two-armed RCT evaluated the effectiveness of *Dojo* in reducing adolescent anxiety. Adolescents with subclinical levels of anxiety played either *Dojo* or the control game *Rayman*. We expected that adolescents who played *Dojo*, relative to *Rayman*, would show reduced anxiety levels at three month follow-up.

Given the importance of understanding moderators of outcomes and the identification of subgroups that are more likely to benefit from an anxiety prevention program [52–54], differential effects for age and sex were also explored. Even though the game is designed for adolescents, there may be differential effects for younger versus older players. Several studies suggest that younger children benefit more from anxiety prevention programs than older children [55,56]. In this line, we expected younger adolescents to report less anxiety symptoms at three month follow-up compared to older adolescents. Furthermore, we tested whether there were any differential effects for sex.

## Materials and Methods

### Design and procedure

Of the 44 eligible secondary schools that were invited to take part in the RCT (i.e., Dutch schools in the province Gelderland within a 1.5 hour travel radius), five schools agreed to participate. We visited the participating schools and during these visits we provided further information about the research project. The recruitment process of participants went through two phases, the screening and the inclusion phase. In the screening phase, we distributed letters about the goals of the study to all 7^th^, 8^th^, and 9^th^ grade students (i.e., students between 11 and 15 years of age) and their parents, in collaboration with the school principals. The letter explained to the parents that they could refuse to have their child participate in the screening. Thus, a passive consent procedure was handled in the screening phase. Initial screening on anxiety levels was conducted among 1,347 adolescents. Screening was performed during school hours in school classes. Adolescents independently completed the Spence Children’s Anxiety Scale (SCAS [57]) in the presence of a teacher and a research assistant. Prior to the screening, adolescents were told that their data would be processed confidentially. There were 349 adolescents who met the inclusion criteria for the RCT (i.e., one standard deviation above the mean on at least two SCAS subscales or on the total SCAS score, with exclusion of the subscale Obsessive Compulsive Disorder). Screening data were collected between November 2013 and May 2014, before the intervention was carried out.

In the inclusion phase, all 349 adolescents and their parents were contacted by phone and informed about the results of the screening and the study goals. Detailed information regarding the results of the screening were only provided to parents with the adolescent’s verbal consent. Adolescents who already received mental health care were excluded from further participation. When an adolescent and his or her parents expressed willingness to participate, they received two information letters (i.e., one for the adolescent and one for the parents) in which the study was explained in full. These letters also explained that adolescents could quit the study at any time and that all gathered information would be treated confidentially. Parents could use the enclosed consent form and return envelopes when both parents and the adolescents agreed to participate. Hence, an active consent procedure was followed during the actual inclusion phase. A total of 138 adolescents (40%) and their parents agreed to participate in the present RCT.

These 138 adolescents were randomly assigned (within schools with an equal gender distribution) to the experimental or the control condition. The experimental group was asked to play *Dojo* and the control group *Rayman*. Adolescents in both conditions played their video game at school and after school hours. They played the game six times over three weeks, with two one-hour sessions per week. These gaming sessions were held in a computer room or in a classroom with individual laptops and headphones, with all adolescents playing in the same room (they could not hear each other or the other games, given the headphones). During all gaming sessions, a research assistant was present. At the first session, the research assistant provided instructions about starting up the game and the rationale of the game for both groups separately. Critically, it was at this first session that adolescents were informed about the condition they were randomly allocated to. At all subsequent sessions adolescents could come in, start the game, and play. The research assistant was present for questions, to monitor the use of headphones, and to maintain silence. All adolescents filled out a digital questionnaire at home at three consecutive time points: prior to the intervention (i.e., pre-test), immediately following the intervention (i.e., post-test), and at a three month follow-up. Pre-test and post-test data were collected between January and July 2014. Follow-up data was collected between May and September 2014. The adolescents received 40 Euros as compensation for their participation in the study. Approval for the design and data collection procedures was obtained from the ethics committee of the Faculty of Social Sciences of the Radboud University Nijmegen (ECSW2013-0410-140). This procedure was also registered before the start of the study in the Dutch Trial Register for RCTs (http://www.trialregister.nl/trialreg/admin/rctview.asp?TC=4379; Trial ID: NTR4379).

### Sample size

Research on indicated anxiety prevention programs generally show small to moderate effect sizes [53,58]. We estimated a small effect of *η*_*p*_^*2*^ = .014 using G*Power 3 [59] with an alpha of .05 and a power of .80. A pre-test/post-test/follow-up (within) by 2 group (between) ANOVA would require 57 adolescents in each study condition leading to a total of 114 adolescents.

### Randomization

Randomization was performed by an independent researcher from the research institute and applied within schools; with an equal number of participants in the control and the experimental condition within one school. Randomization was carried out stratified by sex.

### Participants

A total of 138 adolescents participated in the RCT. At baseline, adolescents ranged from 11 to 15 years old (*M* = 13.27, *SD* = .88) and 35% of the adolescents were male. Nearly all adolescents were born in the Netherlands (97.8%) and the remaining adolescents were born in Turkey, Indonesia, or South Africa (2.2%). Educational levels varied, with 26.1% of the adolescents attending lower general education or lower vocational education, 5.1% attending a combination of lower vocational, lower general education, and higher general education, 9.4% attending higher general education, 10.1% attending a combination of higher general education and pre-university education, and the remaining 49.3% attending pre-university education. Thus, the majority of the sample came from high-streamed education tracks.

### Loss to Follow-up

**Fig 1:**
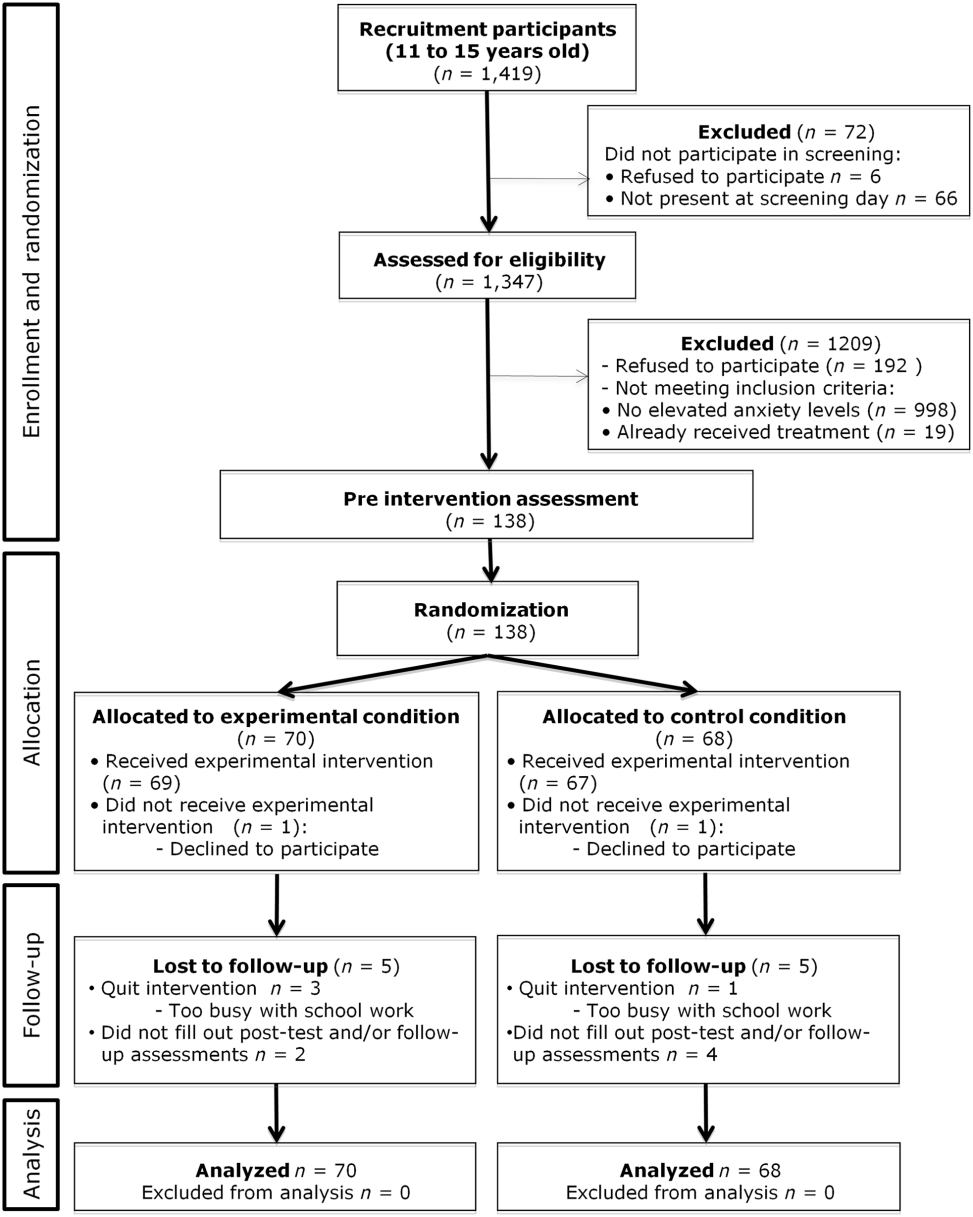
CONSORT Flow diagram.

At pre-test, 138 adolescents took part in the study. The response rate for the post-test was 93.5% (*n* = 129). Six adolescents dropped out during the intervention, and three participants attended the intervention but did not fill out the post-test questionnaire (Fig 1). Of these nine adolescents, five were randomized to the experimental condition and four to the control condition. The response rate for the three month follow-up was 91.3% (*n* = 126). Of the 12 adolescents that did not complete questionnaires, five were randomized to the experimental condition and seven to the control condition. One of the adolescents who did not fill out the post-test questionnaire did fill out the three month follow-up questionnaire. Attrition analyses were conducted to examine if adolescents who stayed in the study and completed the follow-up assessment differed on sex, age, education, ethnicity, study condition, and baseline levels of anxiety symptoms from adolescents who were lost to follow-up. Logistic regression analyses with loss to follow-up as the dependent variable showed no differences on sex (*p* = .765), age (*p* = .733), education (*p* = .311), ethnicity (*p* = .472), study condition (*p* = .828), baseline level of total anxiety symptoms (*p* = .392) and baseline level of personalized anxiety symptoms (*p* = .698).

### Intervention

#### Control condition (Rayman 2: The Great Escape)

*Rayman* is a commercially available platform video game developed by Ubisoft Entertainment S.A. that is played on a PC or laptop. It is played from a third-person perspective, and the player has control over the camera.

*Rayman* was chosen to match the type of attention (adolescents actively playing a video game), but also because it seemed to incorporate game mechanisms that could teach adolescents general skills that may be related to mental health outcomes (e.g., perseverance in the face of failure [60]).

*Rayman* takes place in a world called the Glade of Dreams. Admiral Razorbeard and his army of Robot Pirates have invaded this world thereby greatly weakening Rayman's powers. Rayman learns that in order to stand a chance against the Pirates, he must navigate through the world, defeat numerous threatening enemies, and solve puzzles. Eventually, Rayman succeeds and the Robot Pirates are destroyed.

#### Experimental condition (Dojo)

**Fig 2:**
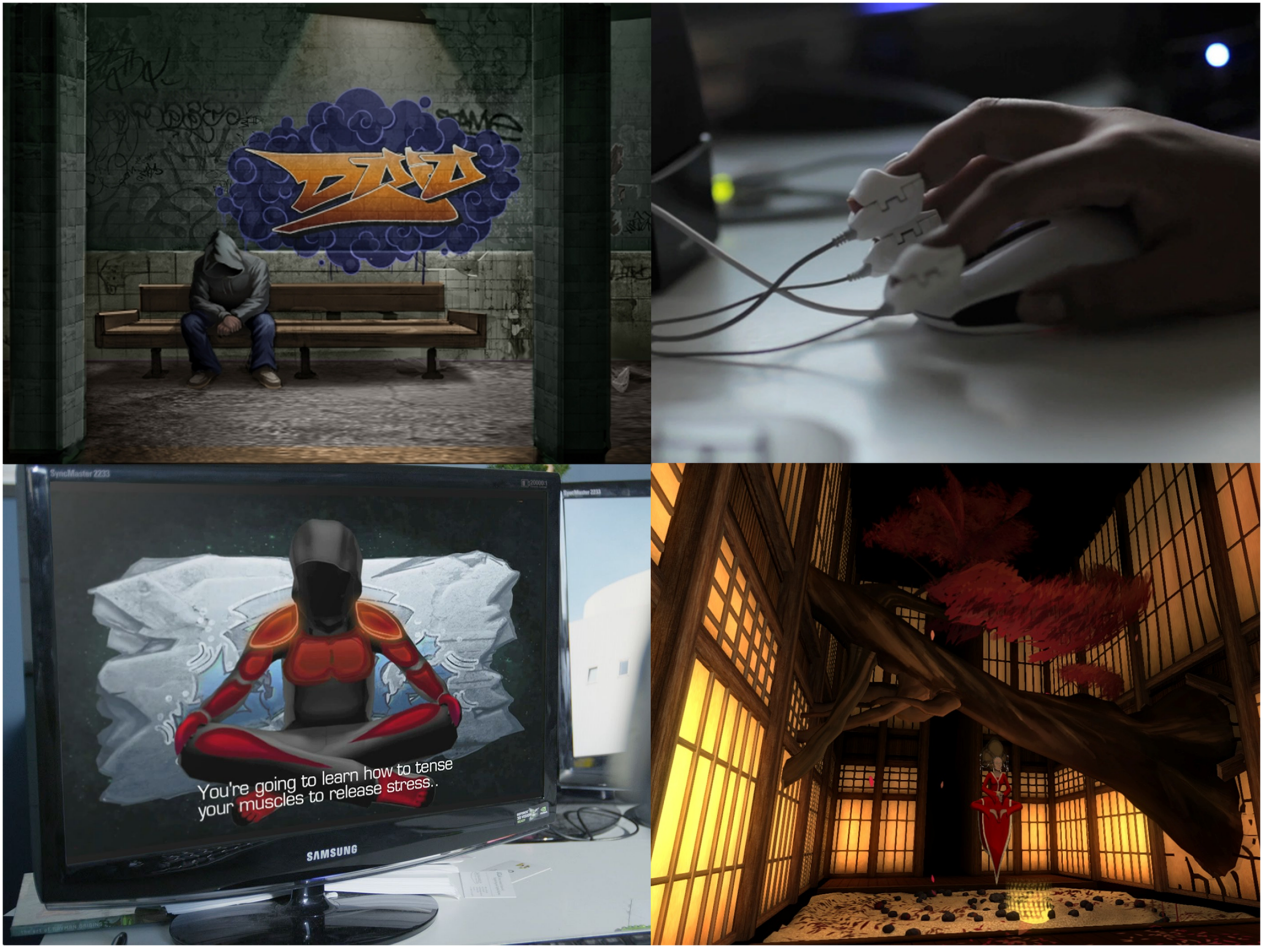
Screenshots *Dojo*.

*Dojo* is a commercially available emotion management biofeedback video game developed by Gamedesk (http://gamedesk.org/project/dojo/; Fig 2) that is played on a PC or laptop. It is played from a first-person perspective.

*Dojo* takes place in a secret temple below an urban subway system with the player experiencing *Dojo* in the role of a young person that is going through a hard time in life. The hidden temple houses three rooms that are thematically designed based on different emotions (i.e., anger, frustration, and fear). In each room, the player encounters a dojo master that instructs the player on emotion regulation strategies, and then presents the player with a challenge that tests the player’s newly acquired emotion regulation skills. In the anger room, the dojo master trains the player in positive self-talk and guided imagery skills; here, the player is challenged to a hand slapping contest where negative sentences (e.g., are you lonely?) pop up on-screen to provoke and distract the player. In the frustration room, the dojo master trains the player in muscle relaxation skills; here, the player is challenged to maneuver a glowing ball through an increasingly complex maze, while not being allowed to touch the maze’s walls. Finally, in the fear room, the dojo master trains the player in deep breathing skills; here, the player is challenged to collect bones in a scary labyrinth and at the same time evade a powerful, frightening, and angered ghost.

During these challenges the player’s heart rate is monitored by a biofeedback system manufactured by Wild Divine that measures heart rate via sensors attached to the player’s fingers (http://www.wilddivine.com/accessories/iom-active-feedback-hardware-by-wild-divine/). Heart rate measures from this system are directly read into *Dojo* so that players’ arousal levels are continuously displayed in the right corner of the video game. To encourage player’s regulation of emotions, each of the game’s challenges become increasingly difficult if the player’s heart rate increases. For example, in one room, the size of the ball which players maneuver through a maze without touching the walls is influenced by the player’s heart rate. The biofeedback system feeds the arousal status of the player back into the game so that if the player gets anxious and his or her heart rate accelerates, the ball becomes bigger making it harder for the player to avoid hitting the maze’s walls. Only by remaining calm and using the emotion regulation strategies taught by *Dojo*’s dojo masters can the player reduce his or her heart rate, diminish the size of the ball, and beat the challenge.

### Measures

#### Game experience

Game experience was assessed at pre-test with the question: “How many hours per week do you play video games?” Adolescents could respond by typing in a number (up to 2 decimals) representing the amount of hours they generally play video games.

#### Game expectations

Expectations about how much playing *Dojo* and *Rayman* could improve anxiety levels were equalized, assessed, and controlled for at pre-test. Before the start of the intervention, all participants read a short overview of both games. Thereby, we intentionally equalized expectations for both games. Participants then indicated on four items whether they thought their real-life behavior could be improved by playing *Dojo* [49]: “Do you think that playing [*Dojo/Rayman*] can help you experience less anxious feelings?”; “Do you think that playing [*Dojo/Rayman*] can help you to be less impulsive?”; “Do you think that playing [*Dojo/Rayman*] can help you to better cope with frustration?”; and “Do you think that playing [*Dojo/Rayman*] can help you to relax more?” Response options were “yes” (= 1) and “no” (= 0). Thereby we primed both groups with the same potential expectations. Both overviews can be found in S1 Appendix.

#### Anxiety

Anxiety was assessed at all time-points with the Spence Children Anxiety Scale (SCAS [57]. The SCAS measures the adolescents’ perception of the frequency in which they experience anxiety-related symptoms (i.e., generalized anxiety, panic/agoraphobia, social phobia, separation anxiety, obsessive compulsive disorder, and fear of physical injury). The SCAS consists of 44 items, 38 of them reflect specific symptoms of anxiety and 6 are positive filler items to reduce negative response bias. Items are answered on a 4-point scale ranging from “never” (= 0) to “always” (= 3). An example item is: “I worry about things”. The SCAS showed good psychometric properties in a Dutch sample [61]. Internal consistency of the 38 specific anxiety symptoms items in the present sample of *n* = 138 was high (*α* = .86). Two outcome variables were calculated based on the SCAS: the mean scale score of the total SCAS (i.e., total anxiety symptoms), and a personalized anxiety symptoms score. This personalized anxiety symptoms score reflects an individual mean of the subscale on which each participant scored highest on at screening. When a participant scored equally high on two or more subscales at screening, a weighted mean of those scales was calculated for each time point.

### Strategy of analysis

We performed Chi-square tests and Analysis of Variance (ANOVA) to examine whether randomization resulted in an equal baseline distribution of relevant participant characteristics across the two conditions. Means and standard deviations were computed for anxiety symptoms at all measurement points separately for both conditions, as were correlations, and independent *t*-tests for sex. Data were analyzed using Mplus 7 [62].

For the main effect analyses, we compared the experimental condition with the control condition. In accordance with the intent-to-treat principle (ITT), all participants that were randomized to a condition were included in these analyses. Missing data were handled by imputing 50 datasets for each condition separately with multiple imputation (MI), using the Markov Chain Monte Carlo (MCMC) method [63]. Linear regression was used with anxiety symptoms (i.e., total anxiety symptoms and personalized anxiety symptoms) as the dependent variable and study condition as independent variable, while controlling for baseline anxiety levels and clustering of data. We used the TYPE = COMPLEX procedure in Mplus to correct for this potential non-independence (complexity) of the data. Between-group and within-group effect sizes (expressed as Cohens’s *d*) were calculated. Potential differential effects of *Dojo* depending on sex were explored by calculating an interaction term between condition (*Rayman* = 0, *Dojo* = 1) and sex (boy = 0, girl = 1) and including this term in the model as a predictor variable. To examine possible differential effects of *Dojo* depending on age, we calculated an interaction term between condition (*Rayman* = 0, *Dojo* = 1) and age. To avoid the problem of multicollinearity, age was centered before computing the interaction term [64]. The interaction term then was included in the model as a predictor variable.

Next, we conducted Latent Growth Curve Modeling (LGCM), including screening, pre-test, post-test and follow-up, to examine the effects of *Dojo* on individual levels of anxiety symptoms at baseline (i.e., the intercept) and changes in anxiety symptoms over time (i.e., the slope [65]). LGCM allows individuals to differ on their starting level of anxiety symptoms and the rate of change in anxiety symptoms over time [65]. To deal with missing data all available pairwise information in the data was used [66]. The Chi-square and *p*-value (*p*-value >.05), the Comparative Fit Index (CFI >.95), and the Root Mean Square Error of Approximation (RMSEA < .05) were applied to assess the goodness of model fit [67].

In the first step of the analysis the growth function (i.e., linear, quadratic, or cubic) that best reflected changes in anxiety symptoms over time was examined for both conditions separately. Anticipating the results by inspecting the means of anxiety symptoms across the four time points, we expected a quadratic growth curve function to fit the data best. Latent growth parameters estimated within a quadratic growth function are: the intercept (*i*), the linear component of the growth curve (*s*), and the quadratic component of the growth curve (*q*). The most appropriate growth function was selected by comparing all functions and interpreting the model fit indices [67]. In a second step, we added condition to the model to test if *Dojo* could predict the initial level, the rate of change, or the curvature of anxiety symptoms. To examine the differences in latent growth parameters between both groups, we estimated and compared a baseline model, an intercept model, a linear model and a quadratic model. If adding new components to the model resulted in a significant increase in chi-square, this would indicate a difference between the control and experimental condition on this component. Finally, we explored if sex or age moderated the relationship between the intervention condition and anxiety symptoms.

## Results

### Descriptive statistics

No differences were observed between the two conditions at baseline for sex, age, ethnicity, education, game expectations, game experience, total anxiety symptoms, and personalized anxiety symptoms (Table 1). Therefore, we did not control for these background variables in our subsequent analyses. Table 2 shows the means and standard deviations for anxiety symptoms at all measurement points, for both conditions separately. The correlations between outcome and background variables are presented in Table 3.

**Table 1:** Comparisons on adolescents baseline characteristics.

	Control	Experimental	Test result
			(Chi-square test or ANOVA)
Sex			
*n* (%)			
Boys	24 (35.3)	24 (34.3)	
Girls	44 (64.7)	46 (65.7)	*Χ*^2^ (1) = .02, *p* = .901
Age			
Mean (SD)	13.83 (.90)	13.90 (.91)	*F*(1,136) = .18, *p* = .677
Ethnicity			
*n* (%)			
Dutch	66 (97.1)	69 (98.6)	
Else	2 (2.9)	1 (1.4)	*Χ*^2^ (1) = .37, *p* = .542
Education			
*n* (%)			
1.	14 (20.6)	22 (31.4)	
2.	5 (7.4)	2 (2.9)	
3.	6 (8.8)	7 (10.0)	
4.	6 (8.8)	8 (11.4)	
5.	37 (54.4)	31 (44.3)	*Χ*^2^ (4) = 3.93, *p* = .416
Game experience			
Mean (SD)	4.89 (5.82)	5.84 (7.97)	*F*(1,136) = .63, *p* = .428
Expectations *Dojo*			
*n* (%)			
ED1 No	29 (43.3)	29 (41.4)	
ED1 Yes	38 (56.7)	41 (58.6)	*Χ*^2^ (1) = .05, *p* = .826
ED2 No	25 (37.3)	29 (41.4)	
ED2 Yes	42 (62.7)	41 (58.6)	*Χ*^2^ (1) = .24, *p* = .622
ED3 No	27 (40.3)	28 (40.0)	
ED3 Yes	40 (59.7)	42 (60.0)	*Χ*^2^ (1) = .00, *p* = .972
ED4 No	26 (38.8)	28 (40.0)	
ED4 Yes	41 (61.2)	42 (60.0)	*Χ*^2^ (1) = .02, *p* = .886
Expectations *Rayman*			
*n*(%)			
ER1 No	44 (65.7)	52 (74.3)	
ER1 Yes	23 (34.3)	18 (25.7)	*Χ*^2^ (1) = 1.21, *p* = .271
ER2 No	42 (62.7)	46 (65.7)	
ER2 Yes	25 (37.3)	24 (34.3)	*Χ*^2^ (1) = .14, *p* = .712
ER3 No	32 (47.8)	33 (47.1)	
ER3 Yes	35 (52.2)	37 (52.9)	*Χ*^2^ (1) = .01, *p* = .942
ER4 No	35 (52.2)	39 (55.7)	
ER4 Yes	32 (47.8)	31 (44.3)	*Χ*^2^ (1) = .17, *p* = .683
Total anxiety symptoms			
Mean (SD)	.86 (.31)	.83 (.33)	*F*(1,136) = .30, *p* = .584
Personalized anxiety symptoms			
Mean (SD)	1.38 (.49)	1.29 (.53)	*F*(1,136) = 1.01, *p* = .318

Education: 1 = lower general education or lower vocational education, 2 = combination of lower vocational and lower general education and higher general education, 3 = higher general education, 4 = combination of higher general education and pre-university education, 5 = pre-university education. ED = expectations *Dojo*, ER = Expectations *Rayman*.

**Table 2:** Means and standard deviations of total anxiety symptoms and personalized anxiety symptoms at all measurement points separately for conditions.

Outcome	Screening	Pre-test	Post-test	Follow-up
Total anxiety symptoms	.97 (.27)	.85 (.32)	.76 (.35)	.71 (.32)
Control	.94 (.27)	.86 (.31)	.78 (.37)	.71 (.35)
Experimental	.99 (.27)	.83 (.33)	.74 (.33)	.72 (.30)
Personalized anxiety symptoms	1.63 (.41)	1.33 (.51)	1.18 (.53)	1.08 (.49)
Control	1.59 (.41)	1.38 (.49)	1.22 (.53)	1.08 (.53)
Experimental	1.67 (.41)	1.29 (.53)	1.13 (.54)	1.08 (.53)

**Table 3:** Correlations among outcome and background variables.

Measures	1.	2.	3.	4.	5.	6.	7.	8.
1. Total anxiety symptoms	1							
2. Personalized anxiety symptoms	.75***	1						
3. Age	-.05	-.02	1					
4. Game	-.17	-.12	-.07	1				
5. Ethnicity	.11	.14	-.07	.29*	1			
6. Education	.06	.08	-.42***	.15	.20	1		
7. ED1	.19*	.10	-.19	-.24*	.10	-.03	1	
8. ER1	.23*	.15	.10	.01	-.22	-.12	.40**	1

*** *p* < .001 level (2-tailed).

** *p* < .01 level (2-tailed).

* *p* < .05 level (2-tailed).

Correlations between variables 1–4 are Pearson correlations and between variables 5–8 Polychoric correlations. Correlations of variables 1–4 with variables 5–8 are Polyserial correlations.

Table 3 shows that adolescents who believed *Dojo* could help them feel less anxious had similar expectations for *Rayman*. Additionally, participants who believed that *Dojo* could help also reported less game experience compared to adolescent who did not believe *Dojo* could help. Finally, adolescents who thought that either *Dojo* or *Rayman* could help them to experience less feelings of anxiety, reported higher levels of total anxiety symptoms at baseline. Independent t-tests for sex and all other variables revealed that boys reported playing video games more often than girls (*t* = 6.53, *p* < .001) and that girls reported higher levels of total anxiety symptoms than boys (*t* = -2.70, *p* = .008).

### Main Effects of *Dojo* on Anxiety Symptoms

For the main effect analyses, the experimental condition (*Dojo)* was compared with the control condition (*Rayman)*. Regression analyses showed that anxiety symptoms significantly decreased at follow-up in both conditions: total anxiety symptoms (*β* = 0.70, SE = 0.04, *p* < .001) and personalized anxiety symptoms (*β* = 0.63, SE = 0.05, *p* < .001). Yet, no prevention effect of study condition at follow-up was found for either total anxiety symptoms (*β* = 0.03, SE = 0.08, *p* = 0.724) or personalized anxiety symptoms (*β* = 0.04, SE = 0.05, *p* = 0.457). The within-group effect sizes for change in total anxiety symptoms from pre-test to post-test was *d* = .25 in the control condition and *d* = .29 in the experimental condition, and from pre-test to follow-up, *d* = .46 in the control condition, and *d* = .39 in the experimental condition. The within-group effect size for change in personalized anxiety symptoms from pre-test to post-test was *d* = .31 in the control condition and *d* = .32 in the experimental condition, and from pre-test to follow-up, *d* = .56 in the control condition, and *d* = .43 in the experimental condition. Between-group effect sizes for change from pre-test to post-test were *d* = -.02 and from pre-test to follow-up *d* = .08 for total anxiety symptoms. For personalized anxiety symptoms, between-group effect sizes were *d* = -.02 for change from pre-test to post-test and *d* = .14 from pre-test to follow-up. Given the small number of drop outs, the completers-only analyses were almost identical to the intention-to-treat results. More detailed information on the completers only analyses can be obtained from the corresponding author. As can be seen from Table 4, no differential effects were observed for sex or age in the relationship between condition and anxiety symptoms.

**Table 4:** Standardized regression coefficients, standard errors and *p*-values of sex and age as moderators in the relation between condition and anxiety symptoms at follow-up.

	Sex			Age		
Outcome	*β*	SE *β*	*p*-value	*β*	SE *β*	*p*-value
Total anxiety symptoms	.068	.147	.644	-.090	.099	.363
Personalized anxiety symptoms	.034	.103	.740	-.032	.131	.808

### Latent Growth Curve Modeling

**Fig 3:**
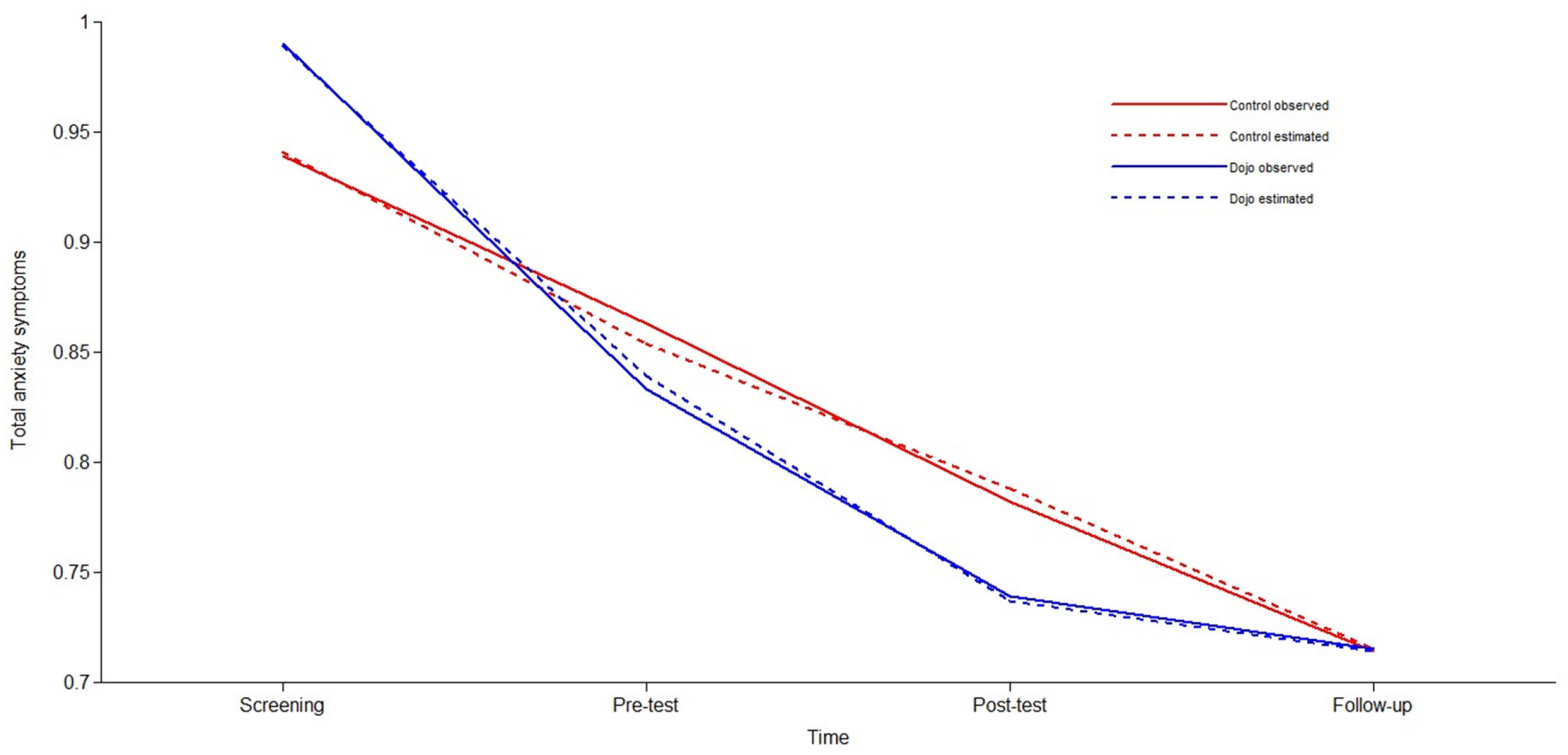
Observed and estimated means for total anxiety symptoms, demonstrating the interaction between time (screening—follow-up) and condition (control/Dojo).

To examine the growth function that best reflected change in anxiety symptoms over time, we first tested linear models with intercept (*i*) and slope (*s*) as latent variables for both conditions separately (Table 5). Since the fit indices were unsatisfactory (*p*-value was < .05, CFI-value was < .95 and RMSEA was > .05), we introduced a quadratic element (*q*) to the model. As can be seen from Table 5, the models including the quadratic component showed an excellent fit for both conditions. For total anxiety symptoms, the parameter estimates for the control condition were *i* = .941 (*p* < .001), *s* = -.098 (*p* < .001), and *q* = .010 (*p* = .014), resulting in the following quadratic function: ANX = .941-.098*T+.010*T^2^ where ANX stands for total anxiety symptoms and T for the months after screening (0, 1, 2, 5). The estimated values of the decrease in total anxiety symptoms for the control condition can be calculated for each time point: .09 (screening—pre-test), .07 (pre-test—post-test), and .08 (post-test—follow-up) (Fig 3). The parameter estimates for total anxiety symptoms in the experimental condition were *i* = .989 (*p* < .001), *s* = -.174 (*p* < .001), and *q* = .024 (*p* < .001) leading to ANX = .989-.174*T+.024*T^2^. The estimated values of the decrease in total anxiety symptoms for the experimental group were: .15 (screening—pre-test), .10 (pre-test—post-test), and .02 (post-test—follow-up).

**Table 5:** Fit statistics: Linear and quadratic model separately for control and experimental condition.

	Control				Experimental			
Outcome	*χ*²(*df*)	*χ*² *p*-value	CFI	RMSEA	*χ*²(*df*)	*χ*² *p*-value	CFI	RMSEA
Total anxiety symptoms								
Linear model	20.48 (6)	.002	.924	.188	48.47 (6)	<.001	.694	.322
Quadratic model	.98 (2)	.611	1.000	.000	1.90 (2)	.387	1.000	.000
Personalized anxiety symptoms								
Linear model	25.64 (6)	<.001	.867	.219	68.49 (6)	<.001	.488	.386
Quadratic model	1.15 (3)	.765	1.000	.000	2.58 (2)	.275	.995	.064

CFI = Comparative Fit Index, RMSEA = Root Mean Square of Approximation

**Fig 4:**
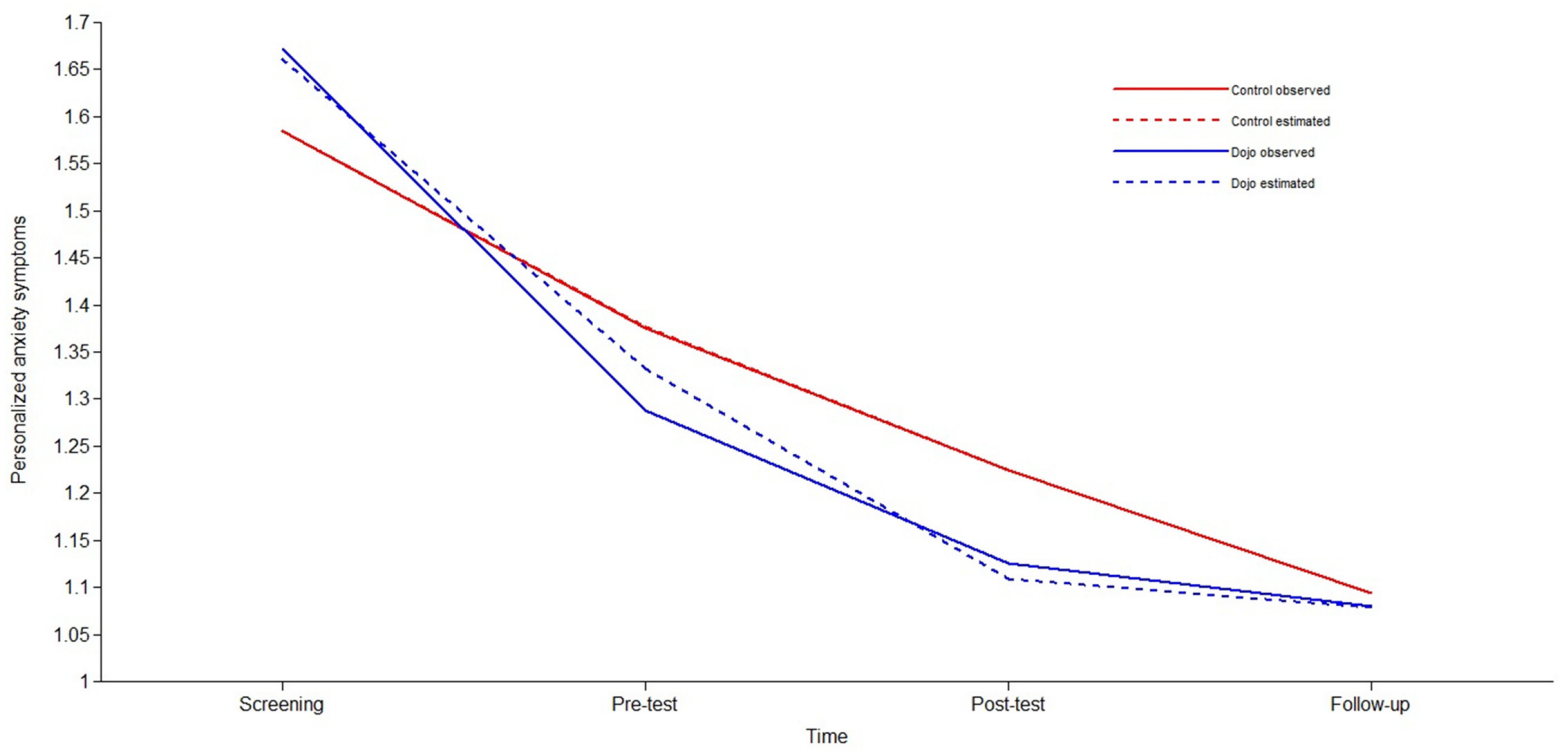
Observed and estimated means for personalized anxiety symptoms, demonstrating the interaction between time (screening—follow-up) and condition (control/Dojo).

The parameter estimates for personalized anxiety symptoms were *i* = 1.585 (*p* < .001), *s* = -.236 (*p* < .001), and *q* = .027 (*p* < .001) for the control condition. The quadratic function was ANX = 1.585-.236*T+.027*T^2^ with ANX being personalized anxiety symptoms and T the months after screening. The estimated values of the decrease in personalized anxiety symptoms were: .21 (screening—pre-test), .16 (pre-test—post-test), and .14 (post-test—follow-up) (Fig 4). The parameter estimates for the experimental condition were *i* = 1.661 (*p* < .001), *s* = -.382 (*p* < .001), and *q* = .053 (*p* < .001), resulting in ANX = 1.661-.382*T+.053*T^2^. The estimated values of the decrease in personalized anxiety symptoms were: .33 (screening—pre-test), .22 (pre-test—post-test), and .03 (post-test—FU).

Finally, we tested the relative effect of *Dojo* on the development of anxiety symptoms over time, by including condition as a predictor in the model (Table 6). To determine the nature of differences between conditions, we compared a baseline model, an intercept model, a linear model, and a quadratic model. The baseline models of both anxiety outcomes showed an excellent fit. No significant differences between the conditions were found for the intercept and the quadratic component in the total anxiety symptoms models (Table 7). The linear component approached statistical significance (*Δχ²*(1) = 3.19, *p* = .074), indicating that there was a marginally steeper linear decrease in total anxiety symptoms observed in the experimental condition compared to the control condition (Fig 3). We did not find differences between conditions in the personalized anxiety symptoms models for the intercept and the quadratic component (Table 7). The linear decrease in personalized anxiety symptoms was significantly higher (*Δχ²*(1) = 5.28, *p* = .022) in the experimental condition (*s* = -.382) than in the control condition (*s* = -.236), indicating that the decrease in personalized anxiety symptoms was significantly steeper for the experimental condition compared to the control condition (Fig 4).

**Table 6:** Initial Level (Intercept), Rate of Change (Slope) and amount of curvature (Quadratic term) in anxiety levels on condition and moderators.

	Intercept	Slope	Quadratic term				
Outcome	*β* (*p*-value)	*β* (*p*-value)	*β* (*p*-value)	*χ*²(*df*)	*χ*² *p*-value	CFI	RMSEA
Condition as predictor							
1. Total anxiety symptoms	.101 (.287)	-.213 (.049)	.234 (.038)	.673 (3)	.888	1.000	.000
2. Personalized anxiety symptoms	.122 (.277)	-.358 (.033)	.372 (.031)	1.773 (3)	.621	1.000	.000
Sex as moderator							
1. Total anxiety symptoms	.260 (.153)	-.064 (.756)	.068 (.753)	.896 (5)	.971	1.000	.000
2. Personalized anxiety symptoms	.378 (.080)	.032 (.907)	-.064 (.818)	2.578 (5)	.765	1.000	.000
Age as moderator							
1. Total anxiety symptoms	-.114 (.402)	.082 (.594)	-.131 (.415)	.743 (5)	.981	1.000	.000
2. Personalized anxiety symptoms	-.119 (.460)	-.037 (.856)	.032 (.874)	1.789 (5)	.878	1.000	.000

CFI = Comparative Fit Index, RMSEA = Root Mean Square of Approximation

**Table 7:** Test results of differences in growth parameters between experimental and control condition.

Outcome	Model	*χ*²(*df*)	*χ*² *p*-value	CFI	RMSEA	*Δχ*²(1)	*p*
Total anxiety symptoms							
	Baseline	2.881 (4)	.578	1.000	.000		
	*i* constrained	4.012 (5)	.548	1.000	.000	1.131	.288
	*i+s* constrained	7.198 (6)	.303	.996	.054	3.186	.074
	*i+s+q* constrained	8.040 (7)	.330	.997	.046	.842	.359
Personalized anxiety symptoms							
	Baseline	2.846 (4)	.584	1.000	.000		
	*i* constrained	4.061 (5)	.541	1.000	.000	1.215	.270
	*i+s* constrained	9.344 (6)	.155	.988	.090	5.283	.022
	*i+s+q* constrained	9.876 (7)	.196	.989	.077	.532	.466

CFI = Comparative Fit Index, RMSEA = Root Mean Square of Approximation

Furthermore, we found no significant interactions for sex or age in the relation between condition and either total anxiety symptoms or personalized anxiety symptoms anxiety (Table 6).

## Discussion

The present study examined the effectiveness of the video game *Dojo* on adolescent anxiety symptoms in a two-armed RCT among an indicated population of 11 to 15 year old adolescents with sub-clinical levels of anxiety. Results showed that adolescent anxiety symptoms significantly decreased in both conditions. Contrary to what we expected, we did not find differences between *Dojo* and the closely matched control condition. We did observe a steeper decrease of personalized anxiety symptoms (not of total anxiety symptoms) in the experimental condition compared to the control condition in our latent growth curve models. Moderation analyses did not show any differences in outcomes between boys and girls nor did age differentiate outcomes.

Surprisingly, we found that participants in both conditions showed equal improvements in anxiety symptoms at follow-up. It could be that neither *Dojo* nor the control condition had an advantageous effect on anxiety symptoms or that both conditions were equally effective. The lack of a no-contact control, such as a wait list, means that we cannot be sure that the decrease in anxiety symptoms was any greater than without an intervention at all. In a meta-analysis, school-based CBT anxiety prevention interventions were compared with wait-list control groups and a mean effect size of *g* = .50 (pre- to post-test) for the school-based CBT prevention interventions and *g* = .19 for the wait-list control group were reported [68]. Comparing our within-group effect sizes with their mean effect size of the wait-list control group, it seems that more improvement was observed in the present RCT than would be expected in an RCT without an intervention as all within-group effect sizes are almost twice the wait-list effect size. Yet, deductions from these indirect comparisons should only be used to stimulate hypotheses for future research and cannot be taken as conclusions.

Even if it is granted that both conditions showed a reduction in anxiety symptoms, it is still unclear why no differences in anxiety symptoms were found between both conditions. There are several explanations for this findings. First, and what we consider most likely, *Rayman*, our control game, may have trained some of the same skills that *Dojo* targets. Even though *Rayman* was not specifically designed for anxiety reduction, it may incorporate some of the more general action mechanisms that have benefits for adolescent’s emotional development more broadly, and anxiety more specifically [22,69]. Video game designers as commercially successful as those who developed *Rayman* are wizards of engagement, they are able to design environments that urge adolescents to work hard toward meaningful goals and celebrate the exceptional moments of success after completing challenging tasks [22]. In *Rayman*, players control a strong and fearless, but also small “underdog” avatar that adolescents can identify with. Players also overcome seemingly insurmountable challenges without instruction or guidance from adults around them. Additionally, this game is built for trial and error learning; most in-game challenges have to be practiced over and over again, and emotions such as anxiety, anger and frustration need to be overcome before players prevail. Through repeated experiences of perseverance in the face of failure [60], perhaps core emotional resilience skills that also relate to anxiety regulation were being trained among our participants. Thus, *Rayman* might have been more than a mere attention control condition by helping to reduce participants’ frustration and anxiety symptoms. We intentionally chose to avoid cluttering the treatment and prevention research literature with a design that used a no-contact control; but by carefully matching our control condition, we are left less clear about the meaning of both groups improving on their anxiety levels [11,49,70]. Future research would benefit from comparing *Dojo* to a control group that is less emotionally stimulating, more focused on “cold” cognitive challenges, and therefore, less related to training skills for regulating anxiety or to an existing more traditional intervention such as regular CBT.

A second explanation for the absence of differences in anxiety symptoms across conditions might be that the duration of the intervention downplayed possible beneficial effects of *Dojo*. In the process evaluation of *Dojo*, adolescents reported that the duration of the intervention (six training sessions) was too long. Specifically, they reported difficulties in maintaining motivation and boredom after approximately four sessions. *Dojo* only includes three rooms with training elements and challenges and most adolescents completed these challenges within three to four sessions. In the remaining sessions these participants had to play the game over and over again, with some reporting frustration with this repetition. Considering that programs with a shorter duration tend to have larger effects and the reported frustration with replaying already completed content suggests that shorter duration play time with *Dojo* might be more effective [71,72].

A last potential explanation might originate in several design-related issues. The intervention was carried out in a group-based context to reach a large number of adolescents. There are studies that report equally strong effects of group-based interventions and individual-based interventions on anxiety [73]. Yet, there are also indications that adolescents with high social anxiety levels respond better to individual interventions [73,74]. The reassurance and social approval provided in an one-on-one context may enhance individual intervention gains. Furthermore, uncontrolled exposure to a group situation may be somewhat overwhelming for socially anxious adolescents, which makes it difficult to fully engage and learn new coping skills [73,74]. As the majority of our sample consisted of adolescents with elevated social anxiety symptoms (70.3%) and multiple adolescents reported issues related to the group context (e.g., not feeling free to fully engage in their video game because of the presence of peers) in their process evaluation, this could have influenced the results.

Another issue concerns the composition of the gaming session groups. Both conditions were randomized within each school and both video games were played in one room simultaneously. Thereby, the control condition could have been contaminated by the experimental condition; adolescents allocated to the control condition may have accidentally received some facets of the intervention since they were in proximity of the experimental group [75,76]. This seems unlikely given that all participants used headphones the entire time they were playing their games; thus, players could not hear what was going on in other games, nor could they talk with one another. Nevertheless, it is recommended for future research to consider whether an individual- or group-based intervention is more applicable and whether two rooms instead of one could be used to deliver the game interventions.

Inspecting the findings on total anxiety symptoms and personalized anxiety symptoms in more detail (Figs 3 and 4), one can see that the largest decrease in anxiety symptoms occurred after screening. This drop in symptoms prior to any intervention is commonly found in studies that report screening to pre-test results [77,78] and is explained by the possible role of non-specific factors in generating intervention effects [79]. Non-specific factors that could have played a role are attention by researchers, the willingness to change, self-monitoring of anxiety symptoms, or emergence of hope [79–81]. We anticipated this phenomenon in both groups because we explicitly and by design attempted to equalize expectations for both groups by framing both *Dojo* and *Rayman* as interventions that could have beneficial effects on mental health. Recall that these expectations were triggered *before* adolescents knew to what condition they were assigned. By doing so, all non-specific factors related to expectations were activated not only in the experimental, but also in the control group.

While non-specific factors could possibly explain the large decrease in anxiety symptoms after screening in both conditions, Figs 3 and 4 also show an observable difference between conditions after screening. Our growth curve models demonstrated that participants in the experimental condition showed a steeper decrease in anxiety symptoms than participants in the control condition between screening and pre-test. This cannot be explained by differences in baseline anxiety symptoms since there were none. Moreover, adolescents’ game expectations prior to randomized assignment were the same across conditions. Although we controlled for expectations, our results still drove a steeper drop in anxiety in the *Dojo* group. Replication and further exploration of possible experimenter effects should be examined in future studies. Moreover, even though participants of both groups had equal expectations before the start of the intervention, it is possible that their expectations changed while playing. For now, however, we argue for the importance of actually attempting to equalize and then adequately measure these expectations at multiple time points and control for them. Otherwise, in our study, we might have interpreted those expectations to be drivers of our differential effects when they clearly were not.

Exploratory analyses did not show moderation effects of sex or age on the relation between condition and anxiety symptoms in our analyses. This finding was consistent over study condition and different sets of analyses; it seems that the games’ effectiveness did not differ between girls and boys, nor for younger versus older adolescents. Although these findings need replication, it is promising to find that video game interventions may appeal equally to boys and girls in different age groups. However, it is also important to note that both games were adventure, puzzle games and the lack of sex and age differences may not hold for different genres of games or diverse mental health concerns [82–84].

### Limitations and implications

A first limitation concerns our use of self-reports. Self-report is necessary to measure subjective anxiety experiences of adolescents in daily life in an ecological valid way, but in addition it is important to use methods that provide insight into the underlying mechanisms of those experiences. Greater physiological reactions to stress (i.e., higher heart rate) and a decreased ability to recover from stressful situations are hallmarks of anxiety [85]. HRV biofeedback video games, such as *Dojo*, could be perfectly suitable to obtain continuously recorded heart rate data of participants during all training sessions and examine heart rate recovery over time. Unfortunately, at this moment the heart rate data recorded by the Wild Divine IOM is hard to interpret because of occasional temporary signal loss [86]. Our recommendation for future research is to incorporate an objective measure of regulated emotional responding and to improve HRV biofeedback materials such as the IOM. HRV does not have to be continuously recorded during interventions; assessments related to real-world anxiety regulation could be equally useful. For example, an assessment of autonomic markers of arousal and regulation before, during, and after a stressful speech task—measured before and after playing *Dojo* for several sessions—could be used for testing whether improvements in psychophysiological responses and recovery mediate intervention success [87].

A second limitation concerns the limited outcome variables that were assessed, given the content of *Dojo* and its training elements. A specific mediating process may be effectively targeted by an intervention, yet if success is primarily evaluated via a broader measure of a disorder (e.g., anxiety), this could result in underestimation of its effectiveness [88]. In this RCT, *Dojo* might target individuals’ anxiety indirectly via coping skills [89]. In future investigations that examine the effects of *Dojo*, it seems important to measure specific coping strategies that are taught in the game, namely adolescents’ tendency to use physiological regulation, relaxation techniques, and self-talk in their lives outside of the game. It seems likely that these strategies may have differentiated *Dojo* from *Rayman*, suggesting separate mechanisms by which the games worked to decrease anxiety. One’s ability to cope with anxious feelings could play a mediating role in the prevention of anxiety. Since we did not include a proper coping outcome measure, we could not test this hypothesis.

## Conclusion

Although we did not find differences between our groups, the effect sizes corresponding to improvements in anxiety symptoms suggest there is promise to a gaming approach. A useful next step may be to develop an extended version of *Dojo* so that it is a longer, more absorbing game that can compete with the high-quality, commercial-grade games on the current market. It also seems important to examine in-game data for both *Dojo* and *Rayman* in order to understand whether certain types of game play mediate outcomes. Although “serious” or “applied” games have been designed for intervention purposes in the medical and education fields, the domain of mental health has been virtually unexplored. We are eager to address this gap by not only designing and improving these games, but also by conducting the most rigorous research on them so that the contribution to prevention science becomes clear and potentially compelling.

## Supporting Information

S1 Appendix Overviews *Dojo* and *Rayman 2*: *The Great Escape*. (PDF)

S1 CONSORT Checklist CONSORT Checklist. (DOC)

S1 Dataset Relevant dataset excluding personal information. (SAV)

S1 Protocol Trial Protocol Dutch. (PDF)

S2 Protocol Trial Protocol English. (PDF)
