# Supplementary material for: A Randomized Controlled Trial to Test the Effectiveness of an Immersive 3D Video Game for Anxiety Prevention among Adolescents
Source: PLoS One. 2016 Jan 27;11(1):e0147763. doi: 10.1371/journal.pone.0147763 (PMC4729475; doi:10.1371/journal.pone.0147763)
Supplement: S1 Appendix — (PDF) [file pone.0147763.s001.pdf]

Overviews *Dojo* and *Rayman 2: The Great Escape*

A description of the contents of a computer game that is tested in this study is given below. Think, while reading the description, about what skills a player should possess to play the video game at best. After the description you get a number of questions in which you can indicate how well you think the video game can help you to learn certain skills.

Dojo

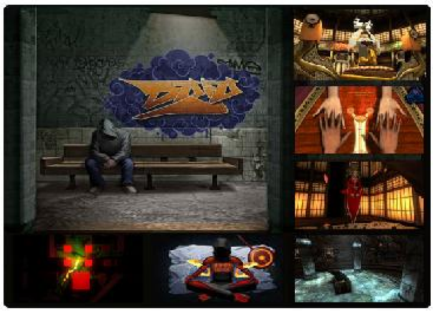

Dojo takes place in a secret temple hidden under the ground. The player will experience the game in the role of a young person who is going to discover this secret temple. In the hidden temple, the player encounters different dojo masters who accompany the player during different trainings and challenges. The player has to face and overcome these challenges.
To overcome the challenges, the player must remain calm in stressful situations.

Rayman 2: The Great Escape

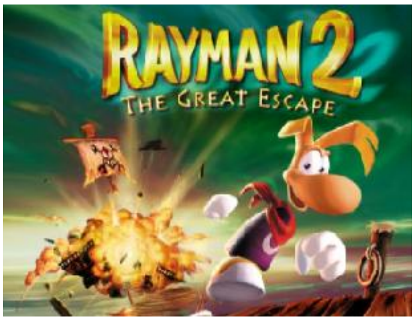

Rayman 2 takes place on a new planet. The player experiences the game in the role of Rayman, a slave. As Rayman, the player must save his planet by going through various challenges and overcome them. To overcome the challenge the player must solve puzzles, defeat enemies and perform under time pressure.
