# Supplementary material for: A Randomized Controlled Trial to Test the Effectiveness of an Immersive 3D Video Game for Anxiety Prevention among Adolescents
Source: PLoS One. 2016 Jan 27;11(1):e0147763. doi: 10.1371/journal.pone.0147763 (PMC4729475; doi:10.1371/journal.pone.0147763)
Supplement: S1 Protocol — (PDF) [file pone.0147763.s004.pdf]

AANMELDINGSFORMULIER ECG

I. ALGEMENE RICHTLIJNEN BIJ HET GEBRUIK VAN DIT FORMULIER

Dit formulier moet voor elk onderzoek worden ingevuld en ingediend bij de facultaire Ethische Commissie Gedragswetenschappelijk onderzoek (ECG). Alleen als er een toelichting gegeven wordt zal de ECG in een vergadering een advies formuleren.

1. Dit formulier kan gebruikt worden voor een individueel experiment (project) of voor een onderzoekslijn. Onderzoekers worden aangemoedigd onderzoekslijnen in te dienen, waarin meerdere experimenten worden voorgesteld die inhoudelijk samenhangen en waarin dezelfde procedures en populaties worden gebruikt. Alleen eerstverantwoordelijke onderzoekers kunnen een protocol indienen, via hun email adres aan de Universiteit te Nijmegen.
2. Ethische goedkeuring van een project of onderzoekslijn is geldig voor een periode van 5 jaar of tot er zich een wijziging voordoet in de hieronder verschafte informatie. Onderzoekers moeten jaarlijks de ECG informeren of het ingediende protocol nog actief is. Wanneer een project of onderzoekslijn is beëindigd, moet de ECG hierover onmiddellijk ingelicht worden.
3. De hieronder vermelde onderzoeker en medewerkers verbinden er zich toe proefpersonen te behandelen volgens de principes van de verklaring van Helsinki (zie ECG-website), en te garanderen dat proefpersonen (wilsbekwaam of –onbekwaam, in een afhankelijkheidsrelatie tot de onderzoeker of niet) te allen tijde medewerking kunnen opzeggen zonder enige gevolgen.
4. De onderzoeker verbindt zich tot het maximaliseren van de kwaliteit van het onderzoek, de statistische analyse en rapportering, en tot het respecteren van specifieke regelgeving in verband met specifieke methodes (vb. fMRI).
5. De onderzoeker verklaart het onderzoek naar waarheid beschreven te hebben, met bijzondere aandacht voor ethische aspecten.

Voor akkoord (Naam in hoofdletters)

hoogleraar	Prof. dr. I. Granic
directeur onderzoeksinstituut	Prof. dr. A.H.N. Cillessen
datum	24Sep2013

II. ALGEMENE INFORMATIE

Titel	Preventie van angst tijdens de adolescentie door het inzetten van een leuke en motiverende video game die gebruik maakt van HRV biofeedback.
Subsidieverstrekker	BSI
Naam projectbegeleider	Isabela Granic
E-mail adres projectbegeleider	i.granic@pwo.ru.nl
Naam projectuitvoerder	Hanneke Scholten
E-mail adres projectuitvoerder	h.scholten@student.ru.nl<br>(attentie: invullen ivm de electronische benaderbaarheid van een eventueel

	concept-formulier)
Locatie(s) van het onderzoek	Middelbare scholen

Vat hieronder het onderzoek samen (maximaal 500 woorden, met accent op beschrijving van procedure, methode en proefpersonen). Indien van toepassing, verstrek voldoende informatie over het gebruik van manipulaties of stimulusmateriaal.

Samenvatting
One of the most popular leisure activities among youth in the United States is playing video games, with 97% of youth between ages 12-17 playing them (Lenhart, Kahne, Middaugh, Macgill, Evans, & Vitak, 2008). In the last five years more research has focused on potential cognitive, social, motivational and emotional benefits for youth provided by video games (Olson, 2010). Consequently, video games could learn youth new forms of strategies, behavior and thought and thereby could be used in a mental health context (Granic, Lobel & Engels, under review). Anxiety disorders are found to be one of the most common adolescent psychiatric disorders and have several negative consequences in different domains, such as interpersonal, emotional or academic functioning (Costello, Egger, Copeland, Erkanli, & Angold, 2011; Pine, 1997). Moreover, anxiety during adolescence is found to persist into adulthood and is related to other serious conditions, such as substance abuse or depression (Cartwright-Hatton, Roberts, Chitsabesan, Fothergill, & Harrington, 2004; Woodward & Fergusson, 2001).The present study will tap into the new area of video game research by testing a video game designed to prevent anxiety in adolescents.

Approximately thousand adolescents between 12 and 14 years of age will be recruited through schools in the region of Nijmegen and will be screened using the Spence Children's Anxiety Scale (SCAS), with passive consent from their parents. Adolescents showing elevated levels of anxiety are asked to participate in the study with active consent from themselves and their parents. Adolescents already receiving mental health care will be excluded from the study.

After screening, 120 participants will be randomly assigned to the experimental or control condition (with equal gender distribution). The experimental group will play the anxiety-reducing video game and the control group a different game for at least 6-8 hours spread over 6 weeks. The experimental game is called “Dojo” and is a challenging, motivating and entertaining video game using emotion regulation strategies (muscle relaxation, breathing techniques, positive self-talk) and HRV biofeedback to reduce anxiety in adolescents. HRV biofeedback will be measured by the Lightstone IOM device, which is non-invasive since the three sensors are attached to three fingertips of the participant. The control game (Super Mario World) is as engaging and difficult as the experimental game, but does not incorporate any anxiety reducing techniques. Participants will fill out the SCAS, Skills and Difficulties Questionnaire and Coping Questionnaire Child before the six week period of video game play, after this period and as a 3-month follow-up. Participants will receive €40,- as a compensation for filling in questionnaires after the 3-month follow-up. The confidentiality of these questionnaires is assured through the use of personal codes, which allow questionnaire data to be stored separate from personal data.

It is hypothesized that, compared to the control group, participants in the experimental condition will show significantly reduced anxiety. Furthermore, we expect that participants in the experimental condition who demonstrate a better understanding of their state of physical arousal will show a larger reduction in anxiety than participants in the experimental condition without or with little understanding of their state of physical arousal.

References
Cartwright-Hatton, S. (2006). Anxiety of childhood and adolescence: Challenges

and opportunities. Clinical Psychology Review, 26, 813–816. doi:10.1016/j.cpr.2005.12.001

Cartwright-Hatton, S., Roberts, C., Chitsabesan, P., Fothergill, C., & Harrington, R. (2004). Systematic review of the efficacy of cognitive behavioural therapies for childhood and adolescent disorders. British Journal of Clinical Psychology, 43, 421-436. doi: 10.1348/0144665042388928

Costello, E. J., Egger, H. L., Copeland, W., Erkanli, A., & Angold, A. (2011). The developmental epidemiology of anxiety disorders: phenomenology, prevalence, and comorbidity. In W.K. Silverman & A.P. Field (Eds.), Anxiety disorders in children and adolescents (pp. 56-75). New York: Cambridge University Press.

Granic, I., Lobel, A., & Engels, C. M. E. (n.d.). The benefits of playing video games. Unpublished manuscript.

Lenhart, A., Kahne, J., Middaugh, E., Macgill, A. R., Evans, C., & Vitak, J. (2008). Teens, video games, and civics. Pew Internet & American Life Project. Retrieved from: http://www.pewinternet.org/Reports/2008/Teens-Video-Games-and-Civics.aspx

Olson, C. K. (2010). Children's motivation for video game play in the context of normal development. Review of General Psychology, 14, 180-187. doi: 10.1037/a0018984

Pine, D. S. (1997). Childhood anxiety disorders. Current Opinion in Pediatrics, 9, 329-339.

Woodward, L. J., & Fergusson, D. M. (2001). Life Course Outcomes of Young People With Anxiety Disorders in Adolescence. Journal of the American Academy of Child & Adolescent Psychiatry, 40, 1086-1093. doi: 10.1097/00004583-200109000-00018

Heeft u dit of soortgelijk onderzoek al eerder ingediend bij de ECG?

(x) Nee
( ) Ja, ECG-nummer

Betreft dit een onderzoeksproject of een onderzoekslijn? Onderzoeksproject

III. ETHISCHE RICHTLIJNEN

1. Hieronder staan een aantal regels waaraan volgens de ECG voldaan moet worden. Het navolgen van deze regels is de verantwoordelijkheid van de individuele onderzoeker of diens supervisor. Wordt aan de onderstaande regels (A-D) voldaan?

A. Het onderzoek zal naar verwachting nieuwe en belangrijke inzichten opleveren.
B. Het onderzoek voldoet aan de eisen van juiste methodologie.
C. Het onderzoek geschiedt onder supervisie van een deskundig persoon. Diegene die mogelijke ingrepen verricht dient deskundig en ervaren te zijn.
D. Het onderzoek vindt niet plaats met proefpersonen die buiten het onderzoek in een ondergeschikte positie ten aanzien van de onderzoeker verkeren (bv. eigen kinderen).

(x) Ja
( ) Nee, toelichting:

2. Is er een aanspreekpunt waar proefpersonen terecht kunnen met vragen over het onderzoek en worden zij hiervan op de hoogte gesteld?

(x) Ja
( ) Nee, toelichting:

3. Is duidelijk waar klachten over deelname aan het onderzoek kunnen worden geuit en hoe deze behandeld zullen worden?

(x) Ja
( ) Nee, toelichting:

4. Zijn de proefpersonen volledig vrij om deel te nemen aan het onderzoek, en om hiermee op elk moment te stoppen wanneer zij dat willen, om welke reden dan ook?

(x) Ja
( ) Nee, toelichting:

5. Worden proefpersonen en/of hun vertegenwoordiger voor deelname voorgelicht over doel, aard en duur, risico's en bezwaren van de studie, en tekenen zij voor deelname middels de standaard informed consent (zie de formulieren op de website)?

Het is standaard dat proefpersonen of hun wettelijke vertegenwoordiger

a. vooraf schriftelijk en mondeling over de aard van het onderzoek volledig zijn ingelicht
b. schriftelijke toestemming geven via het gepaste Toestemmingsformulier
c. terugkoppeling: achteraf schriftelijk en mondeling in kennis worden gesteld van de doelstelling van het onderzoek

De voorlichting is duidelijk en afgestemd op de proefpersoon (bijvoorbeeld ingeval van minderjarigen). De proefpersoon krijgt bedenktijd tussen informatie en toestemming. Indien volledige informatie vooraf onmogelijk is vanwege de aard van het onderzoek, dient de proefpersoon achteraf te worden geïnformeerd. Ook minderjarige en wilsonbekwame proefpersonen moeten in alle gevallen toch naar bevattingsvermogen worden geïnformeerd.

( ) Ja
(x) Nee, toelichting:
Scholen die willen deelnamen aan het onderzoek krijgen voordat de screening plaatsvindt brieven toegestuurd waarin alle participanten en ouders/verzorgers worden

geïnformeerd over het onderzoek. In deze brieven wordt toestemming gevraagd aan de ouders/verzorgers voor deelname van hun kind aan de screening door middel van passief consent. Aan de ouders zal aangegeven worden dat de kinderen gescreend worden op een aantal factoren om deel te mogen nemen aan het onderzoek en dat hun kind mogelijk wordt uitgenodigd om mee te doen aan het vervolg van het onderzoek. Middels passieve consent hebben ouders de mogelijkheid om deelname van hun kind te weigeren. Ouders hoeven dus niet expliciet voor toestemming te tekenen voor de screening. Dit heeft verschillende redenen:
a) In eerder onderzoek naar de effecten van de depressie preventie programma's 'Op Volle Kracht' en 'Sparx' (A randomized controlled trial comparing school-based (Op Volle Kracht) and computerized (SPARX) depression prevention programs with adolescent girls, uitgevoerd door Marlou Poppelaars) is gebleken dat ouders tevreden zijn over het toepassen van passieve consent. Bovendien hebben scholen toen ook hun voorkeur uitgesproken voor passieve consent omdat ouders dan toch de mogelijkheid hebben om geen toestemming te geven voor deelname voor hun kind.
b) Het voorkomt vertraging in het onderzoek en hoge non-respons van ouders/verzorgers.

Aan participanten die geselecteerd worden voor het onderzoek zal telefonisch toestemming gevraagd worden om deel te nemen aan het vervolg van het onderzoek. Ook aan ouders zal op dit punt in het onderzoek expliciet toestemming worden gevraagd voor deelname van hun kind aan het verdere onderzoek.

6. Indien er misleiding plaatsvindt, voldoet de procedure dan aan de standaardeisen (geen misleiding over risico's, accurate debriefing)?

Onder misleiding wordt verstaan het verschaffen van inaccurate of onvolledige informatie aan de proefpersoon. Misleiding is slechts toegestaan als er geen mogelijkheid bestaat de vraagstelling zonder misleiding te beantwoorden. Misleiding is niet toegestaan als het gaat om informatie over de eventuele risico's die verbonden zijn aan deelname. Na misleiding vindt er altijd een volledige debriefing van de proefpersoon plaats over de manier waarop de proefpersoon is misleid. Indien er redelijkerwijs tijdelijke negatieve effecten van een misleiding zijn te verwachten, dan vindt deze debriefing plaats onmiddellijk na het beëindigen van het experiment (bijvoorbeeld als er valse negatieve feedback wordt gegeven over intelligentiescores, dan vindt er onmiddellijke debriefing plaats). De debriefing is op zo'n manier gesteld dat redelijkerwijs mag worden verwacht dat de tijdelijke negatieve effecten op bijvoorbeeld zelfbeeld en stemming door de debriefing worden weggenomen. Indien geen tijdelijke negatieve effecten worden verwacht mag de debriefing ook op een later tijdstip plaatsvinden, echter uiterlijk binnen een maand na het beëindigen van het experiment.

(x) Er vindt geen misleiding plaats
( ) Er vindt misleiding plaats die geheel voldoet aan de eisen
( ) Er vindt misleiding plaats die niet voldoet aan de standaardeisen (toelichting)

7. Wordt voldaan aan de standaard regels in verband met anonimiteit en privacy?

De onderzoeksgegevens worden standaard

a. anoniem verwerkt en vertrouwelijk opgeslagen
b. met inzagerecht voor de proefpersoon van de eigen gegevens
c. met inzage van alle data voor alle onderzoekers betrokken bij het project

(x) Ja
( ) Nee, toelichting:

IV. DE PROEFPERSONEN

8. Gaat het om een gezonde populatie?

(x) Ja
( ) Nee, toelichting:

9. Worden de proefpersonen gescreend teneinde de risico's voor nadelige effecten van het onderzoek te reduceren?

(x) Nee
( ) Ja, toelichting:

10a. Is er sprake van onderzoek bij minderjarigen (<18 jaar) of bij wilsonbekwamen?

Wilsonbekwamen zijn personen die niet in staat zijn tot een redelijke waardering van hun belangen ter zake. (Zie de notitie 'nee tenzij')

(x) Ja, door met vraag 10b
( ) Nee, door met vraag 11

10b. Gaat het om doelgroepgebonden onderzoek?

Doelgroepgebonden onderzoek is onderzoek dat niet zonder deelname van de groep waartoe de proefpersoon behoort kan worden uitgevoerd, bv. minderjarigen, dementerende ouderen. (Zie de notitie 'nee tenzij')

(x) Ja
( ) Nee, toelichting:

10c. Is er een praktische reden waarom minderjarigen of wilsonbekwamen aan het onderzoek meedoen, bv. omdat er onvoldoende wilsbekwame personen geworven kunnen worden? (Zie de notitie 'nee tenzij')

(x) Nee
( ) Ja, toelichting:

10d. Is het mogelijk om de benodigde kennis eerst bij volwassen, wilsbekwame personen te verkrijgen?

(Zie de notitie 'nee tenzij')

(x) Nee
( ) Ja, toelichting:

10e. Is er sprake van therapeutisch onderzoek, d.w.z. dat de studie mede ten goede kan komen aan de proefpersoon zelf?

(Zie de notitie 'nee tenzij')

(x) Ja
( ) Nee, toelichting:

V. DE GEHANTEERDE METHODE

11. Wordt er een methode gebruikt die het mogelijk maakt bij toeval een bevinding te doen waarvan de proefpersoon op de hoogte zou moeten worden gesteld?

Sommige methoden van onderzoek kunnen toevalsbevindingen opleveren die van belang kunnen zijn voor de proefpersoon. Te denken valt aan hartritmestoornissen op een ECG, een afwijkend EEG (epilepsie), of een afwijking op een fMRI. Indien die mogelijkheid bestaat dient in het informed consent een bepaling te worden opgenomen die voorziet in de dan te volgen procedure. De proefpersoon dient bij die onderzoeken hetzij de naam en praktijkplaats van haar/zijn huisarts dan wel het complete praktijkadres van de huisarts of huisartsenpraktijk op te geven, die wordt verwittigd in het geval van een voor haar/hem van belang zijnde bevinding. Heeft de proefpersoon geen huisarts dan dient zij/hij akkoord te gaan met het feit dat de studentenarts, of in voorkomende gevallen een bedrijfsarts wordt verwittigd. De proefpersoon dient met deze procedure akkoord te gaan door middel van het ondertekenen van een aparte clausule op het informed consent formulier.

( ) Nee
(x) Ja, toelichting:
Tijdens het spelen van de video game Dojo zullen de participanten zijn aangesloten op een IOM HRV biofeedback systeem. Dit systeem geeft echter geen volledig overzicht van het ECG van de participant, maar een getal in een bepaalde range als weergave van de beats per minute (BPM). Omdat de output te weinig informatie verstrekt over het ECG van de participant, kunnen er geen mogelijke hartritmestoornissen of andere hartafwijkingen aan het licht komen.

12. Wordt er gebruik gemaakt van unobtrusive methoden?

Bij unobtrusive methoden worden data verzameld, zonder dat de proefpersoon hiervan op de hoogte is. Het gedrag van de proefpersoon wordt bv. geobserveerd of op video opgenomen zonder dat deze het weet.

(x) Nee
(   ) Ja, toelichting:

13. Worden proefpersonen aan handelingen onderworpen of wordt aan de proefpersonen een bepaalde gedragswijze opgelegd, die buiten de normale levenswijze vallen?

Voorbeelden: stress opwekken, druk uitoefenen om belangrijke normen en waarden te overschrijden, valse herinneringen induceren, verwerking van ernstige gebeurtenissen, langdurige of zeer frequente ondervraging, ambulante metingen, effectiviteit van interventies, onaangename psychische of fysieke symptomen oproepen binnen een experiment (Zie de notitie gedragswetenschappelijk onderzoek en de WMO)

(   ) Nee
(x) Ja, toelichting:
In dit onderzoek wordt de effectiviteit van twee video games getest in de preventie van angst. Deze video games zijn voornamelijk gericht op alledaagse stressoren en angstige situaties voor adolescenten. Er zal geen sprake zijn van het bewust opwekken van stress, druk uitoefenen om belangrijke normen en waarden te overschrijden, het induceren van valse herinneringen, verwerking van ernstige gebeurtenissen, van langdurig of zeer frequent ondervragen, ambulante metingen, effectiviteit van interventies. Het is echter mogelijk dat de video game angst, en daarbij onaangename psychische of fysieke symptomen, kan oproepen bij de adolescent, maar de video game biedt daarbij ook handvatten om deze angst en onaangename symptomen te overwinnen. De trainer zal de participanten nauwkeurig in de gaten houden en eventuele signalen van te heftige angst zullen worden besproken met de projectuitvoerder.

14. Zijn de risico's en bezwaren minimaal? Is het risico dat men loopt een risico dat men in het dagelijks leven ook loopt?

(x) Ja
(   ) Nee, toelichting:

15. Wordt er een andere dan de standaardvergoeding geboden aan de proefpersonen?

(x) Nee
(   ) Ja, toelichting:

VI. AANVULLENDE INFORMATIE

Gebruik de hieronder voorziene ruimte voor aanvullingen of informatie waarvoor er op dit formulier onvoldoende ruimte voorzien was.

VII. TOEGEVOEGDE FORMULIEREN

Samen met dit formulier moeten de volgende documenten worden ingediend:

- de tekst van de advertenties;
- de tekst van de schriftelijke terugkoppeling (debriefing) met vermelding contactinformatie van verantwoordelijke onderzoeker;
- een kopie van het toestemmingsformulier;
- voor onderzoek op een externe locatie (buiten het Spinozagebouw), waar proefpersonen vallen onder de verantwoordelijkheid van een externe instelling (bv. school, ziekenhuis), moet een toestemmingsverklaring ondertekend door deze instelling bijgevoegd worden.[1]

Radboud Universiteit Nijmegen

Faculteit der Sociale Wetenschappen

Radboud Universiteit Nijmegen

T.a.v. Hanneke Scholten

Orthopedagogiek Gezin & Gedrag, A.06.11a

Montessorilaan 3

6525 HR Nijmegen

Tel: 024-3612551

Fax: 024-3612776

Email: h.scholten@pwo.ru.nl

www.ru.nl/fsw

Telefoonnummer	Datum
024-3612551	23 oktober 2013

Betreft	E-mail
Uitnodiging deelname weerbaarheidstudie	h.scholten@pwo.ru.nl

Beste meneer/mevrouw,

Zoals u op de flyer kunt lezen zijn wij bezig met een onderzoek naar twee video games die worden geëvalueerd op hun effectiviteit. In dit onderzoek richten wij ons specifiek op het weerbaar maken van jongens en meisjes tijdens hun adolescentie. We zullen onderzoeken welke van de twee video games effectiever is in het aanleren van bepaalde strategieën aan jongeren, zoals doorzettingsvermogen, regulatie van emoties zoals angst, boosheid of frustratie en het omgaan met tegenslagen en falen. Wij sturen u deze flyer en brief met het verzoek om aan ons onderzoek deel te nemen.

Op scholen die deelnemen aan het onderzoek zullen wij in november of december 2013 een vragenlijst afnemen bij alle jongens en meisjes van het eerste en tweede jaar. Deze vragenlijst gaat o.a. over hoe de jongeren denken, wat ze voelen en hoe ze met problemen omgaan. Vervolgens zullen de jongeren worden geselecteerd om deel te nemen aan het onderzoek.

Beide video games zullen door de jongeren die geselecteerd zijn op school maar na schooltijd of in tussenuren worden gespeeld. De jongeren zullen 6 uur verspreid over 3 weken (in de periode tussen januari en april 2014) één van beide video games spelen. Voorafgaand aan en na afloop van de 3 weken vullen de jongeren een aantal vragenlijsten in gericht op moeilijkheden die ze tegenkomen in het dagelijks leven en de manieren waarop ze daar mee om weten te gaan. Vervolgens wordt er 3 maanden na afloop van de 3 weken na schooltijd of in tussenuren een vragenlijst afgenomen om de veranderingen en effecten op de langere termijn te kunnen bepalen. Iedere deelnemende jongere ontvangt een vergoeding van €40,- als tegenprestatie voor het invullen van de vragenlijsten.

Wij hopen van harte dat u wilt deelnemen aan het onderzoek aangezien het een belangrijke bijdrage kan leveren aan de bevordering van de mentale gezondheid van Nederlandse jongeren. Als dank voor uw deelname bieden we na afloop van het onderzoek een rapport aan over de uitslag van het onderzoek. Ook bestaat de mogelijkheid deskundigheidsbevordering voor docenten te organiseren.

Wij realiseren ons dat het begin van het schooljaar druk is en houden deze brief dan ook bewust kort. Graag horen wij van u of u mee wil werken aan de weerbaarheidstudie. Wij bellen u hierover binnen een week terug. U kunt hiervoor ook zelf contact opnemen via de mail (h.scholten@pwo.ru.nl), of bellen naar 024-3612551. Natuurlijk kunt u hier ook terecht voor vragen of meer informatie.

Namens het hele projectteam dank ik u hartelijk voor uw aandacht en een eventuele reactie,

Hanneke Scholten

Projectteam:

Prof. Dr. Isabela Granic (Radboud Universiteit Nijmegen)

Prof. Dr. Rutger Engels (Radboud Universiteit Nijmegen)

Adam Lobel, Phd student (Radboud Universiteit Nijmegen)

Hanneke Scholten Bsc. (Radboud Universiteit Nijmegen)

Radboud Universiteit Nijmegen

Onderzoek naar effectiviteit weerbaarheidtrainingen op middelbare scholen

Geachte ouder(s)/verzorger(s),

Met deze brief willen wij u graag informeren over het verdere verloop van het onderzoek naar de effectiviteit van twee weerbaarheidtrainingen voor jongeren met verminderde weerbaarheid en assertiviteit.

Zoals u weet vindt het Over Betuwe College Huissen dit onderzoek een meerwaarde voor de zorg voor haar leerlingen en heeft ingestemd met het uitvoeren hiervan. Ook de ethische commissie van de Faculteit der Sociale Wetenschappen van de Radboud Universiteit heeft haar goedkeuring gegeven voor de uitvoering van dit onderzoek. U heeft eerder al informatie ontvangen over het onderzoek en heeft toegestemd met deelname van uw kind aan de screening van het onderzoek. Met deze brief willen wij u informeren over de uitkomsten van deze screening en het verdere verloop van het onderzoek.

Waarom dit onderzoek?

Onzekerheid, verminderde mentale weerbaarheid en verminderde assertiviteit zijn problemen waar behoorlijk wat jongeren mee te maken hebben en die van grote invloed kunnen zijn op hun functioneren. Er is steeds meer bewijs dat video games een positieve invloed kunnen hebben op sociale, cognitieve en emotionele vaardigheden van jongeren. Daarnaast hebben video games als grote voordeel dat ze jongeren kunnen motiveren. Deze voordelen van video games hebben de potentie om jongeren weerbaarheidstrategieën aan te leren in een uitdagende context. Omdat het inzetten van video games in het weerbaar maken van jongeren een zeer nieuwe aanpak is, hebben wij uw zoon of dochter en u nodig om deze video games te evalueren. Daarnaast willen we met dit onderzoek bekijken hoe effectief beide trainingen zijn in het helpen van jongeren met milde klachten aangaande weerbaarheid, assertiviteit en onzekerheid.

Resultaat van de screening

Zoals u weet heeft uw kind deelgenomen aan de screening van het onderzoek. De screening hield in dat alle jongeren uit de tweede klas een vragenlijst hebben ingevuld. Met behulp van deze vragenlijst wilden we bekijken in welke mate uw kind klachten ervaart op het gebied van onzekerheid, assertiviteit en weerbaarheid. Zoals aan de telefoon ook al is besproken, bleek uit deze vragenlijst dat uw kind meer dan leeftijdsgenoten aangeeft problemen te ervaren op deze gebieden.

Wat houden de weerbaarheidtrainingen in?

Dit onderzoek richt zich op twee verschillende weerbaarheidtrainingen in de vorm van video games, “Dojo” en “Rayman 2: The Great Escape”. Beide video games zijn erop gericht jongeren bepaalde

strategieën aan te leren die ze kunnen helpen om meer weerbaar te worden. Vaardigheden als doorzettingsvermogen, omgaan met tegenslagen of falen en omgaan met emoties zullen aan bod komen in beide weerbaarheidtrainingen.

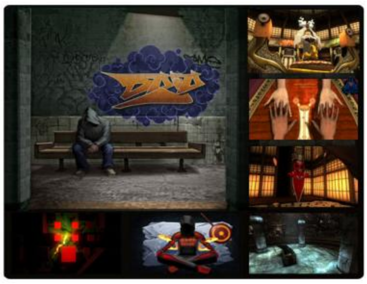

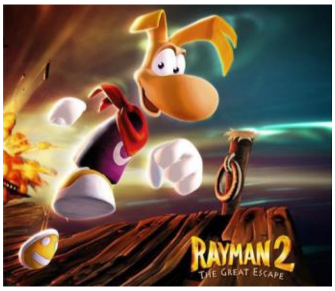

<u>Links</u>

beelden uit de “Dojo” video game

<u>Rechts</u>

beelden uit de “Rayman 2: The Great

Wij willen benadrukken dat beide video games geen therapeutische programma’s zijn, en zeker geen zwaarmoedige programma’s die zich richten op zware problemen. We geloven dat juist de inzet van luchtige, leuke en motiverende video games effectiever zal zijn in het weerbaar maken van jongeren.

Het verdere onderzoek

Omdat uw kind aangeeft meer dan gemiddeld klachten te ervaren van onzekerheid, assertiviteit en weerbaarheid, komt uw kind in aanmerking om deel te nemen aan het verdere onderzoek. Dit houdt in dat uw kind kan deelnemen aan één van de twee weerbaarheidtrainingen. Jongeren die op dit moment al in behandeling zijn voor dit soort klachten, kunnen helaas niet deelnemen aan het verdere onderzoek. De weerbaarheidtrainingen bestaan beide uit 6 sessies van een uur verspreid over drie weken. Deze sessies zullen op school, maar na schooltijd plaatsvinden. De weerbaarheidtrainingen zijn kosteloos.

Alle deelnemende jongeren wordt gevraagd om drie keer een vragenlijst in te vullen over hun angsten, zorgen en hun vaardigheden om daarmee om te gaan: in februari 2014, maart 2014 en juni 2014. Deze vragenlijsten ontvangt uw kind per mail en het invullen van de vragenlijsten zal per keer ongeveer 30 minuten in beslag nemen. Indien uw kind op alle drie de tijdsmomenten alle vragenlijsten heeft ingevuld en deelgenomen heeft aan alle zes trainingssessies (met uitzondering van afmelding met een goede reden zoals ziekte) ontvangt uw kind een vergoeding van 40,- euro.

De deelnemende jongeren worden willekeurig verdeeld over twee groepen. De ene helft van de jongeren zal het spel “Dojo” gaan spelen en de andere helft zal het spel “Rayman 2: The Great Escape” gaan spelen. Omdat de verdeling willekeurig is, hebben wij geen invloed op welke jongeren in welke groep worden geplaatst.

Vertrouwelijkheid

We willen graag benadrukken dat *alle* gegevens vertrouwelijk zullen worden behandeld en dat in geen enkel rapport namen vermeld zullen worden. Uitsluitend de onderzoekers van de universiteit hebben inzage in de vragenlijst. Bovendien worden de gegevens **nooit** aan derden verstrekt. Uiteraard zijn u en uw kind niet verplicht om deel te nemen aan het onderzoek.

Hoe verder?

Als bijlage bij deze brief vindt u de toestemmingsverklaring en een retourenvelop. Indien u toestemt met deelname van uw kind aan het verdere onderzoek, dient u deze toestemmingsverklaring in te vullen en te ondertekenen en uiterlijk 7 februari 2014 terug te sturen in de bijgevoegde retourenvelop. Nogmaals, u en uw kind zijn niet verplicht om deel te nemen. U kunt op elk moment stoppen met het onderzoek.

Wij hopen u met deze brief voldoende te hebben geïnformeerd. Wanneer u nog inhoudelijke vragen heeft over het onderzoek, aarzelt u dan niet om contact op te nemen met Hanneke Scholten (024-3612551). U kunt ook een e-mail sturen naar h.scholten@pwo.ru.nl.

Dank voor uw aandacht!

Vriendelijke groeten namens het projectteam,

Hanneke Scholten

Projectteam:

Prof. Dr. Isabela Granic (Radboud Universiteit Nijmegen)

Prof. Dr. Rutger Engels (Radboud Universiteit Nijmegen)

Adam Lobel, Phd student (Radboud Universiteit Nijmegen)

Hanneke Scholten Bsc. (Radboud Universiteit Nijmegen)

Radboud Universiteit Nijmegen

Omgaan met lastige situaties, hoe doe je dat?
Een onderzoek op jouw school.

Beste leerling,

Op jouw school is een tijdje geleden een vragenlijst afgenomen bij alle leerlingen van de tweede klas. Daarbij is toen verteld dat een aantal leerlingen opgebeld zouden worden met de vraag of ze mee willen doen aan de rest van het onderzoek. Met deze brief willen we je graag wat vertellen over het onderzoek waarvoor wij deze vragenlijst hebben afgenomen en hoe het vervolg van het onderzoek er uit gaat zien.

Waarom dit onderzoek?
Als je in de leeftijd van 12 tot 14 jaar bent, kom je veel uitdagingen tegen. Je gaat bijvoorbeeld van de basisschool naar de middelbare school, je leert allemaal nieuwe mensen kennen, je probeert nieuwe vrienden te maken en je probeert goede cijfers te halen. Omdat er in deze periode zoveel dingen gebeuren, is het niet raar dat sommige jongeren dit af en toe lastig vinden. Sommige jongeren zijn bijvoorbeeld ontevreden over hun uiterlijk, anderen voelen zich soms alleen en een aantal jongeren is bang voor verschillende dingen. Omdat deze problemen best vaak voorkomen in de leeftijd van 12 tot 14 jaar worden er zogenaamde weerbaarheidtrainingen ontwikkeld die jongeren kunnen helpen om beter in hun vel te komen zitten. Weerbaarheidtrainingen leren jongeren om minder onzeker te zijn, voor zichzelf op te komen en zich beter te voelen. Wij hebben ervoor gekozen om twee video games te gebruiken als weerbaarheidtrainingen, namelijk “Dojo” en “Rayman 2: The Great Escape”. Omdat video games nog nooit gebruikt zijn om jongeren weerbaar te maken, hebben we jou nodig om te testen of video games daarvoor geschikt zijn. Wat jij van de video game vindt is dus heel belangrijk!

De uitkomst van de vragenlijst
Wij willen, als onderzoekers van de Radboud Universiteit, graag onderzoeken of video games ervoor kunnen zorgen dat jij weerbaarder wordt. Om dit te kunnen onderzoeken, hebben we eerst een vragenlijst afgenomen bij alle leerlingen van het derde jaar. Deze vragenlijst ging over hoe je denkt, wat je voelt en hoe je met moeilijkheden omgaat. Zoals we je aan de telefoon ook al hebben verteld, bleek uit de vragenlijst dat jij je soms wat meer zorgen maakt of wat banger bent voor sommige dingen dan je leeftijdsgenoten.

Wat houden de weerbaarheidtrainingen in?
Dit onderzoek richt zich op twee verschillende video games, “Dojo” en “Rayman 2: The Great Escape”. Beide video games leren je om door te zetten in een lastige situatie, om te gaan met tegenslagen en om te gaan met verschillende emoties zoals angst of boosheid.

Hoe ziet het onderzoek er precies uit?
Als je mee wilt doen aan het onderzoek, zul je drie weken lang twee keer per week één van de twee video games spelen. Dit zijn in totaal zes sessies die allemaal een uur duren. Voordat je gaat beginnen met het spelen van de video game, na afloop van de zes sessies en drie maanden na afloop

van de zes sessies zullen we jou vragen om een paar vragenlijsten in te vullen. Met de uitkomsten van die vragenlijsten kunnen wij testen of “Dojo” en/of “Rayman 2: The Great Escape” jongeren kunnen helpen om meer weerbaar te worden. Je wordt willekeurig ingedeeld in één van de twee groepen. De eerste groep zal het spel “Dojo” gaan spelen en de tweede groep zal het spel “Rayman 2: The Great Escape” gaan spelen. Omdat de verdeling willekeurig is, weten we niet in welke groep je terecht zal komen. Als je meedoet aan alle zes sessies en op alle drie de tijdsstippen alle vragenlijsten hebt ingevuld zul je een beloning van 40 euro ontvangen.

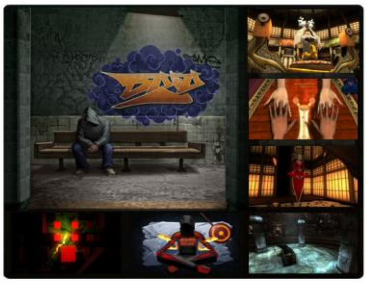

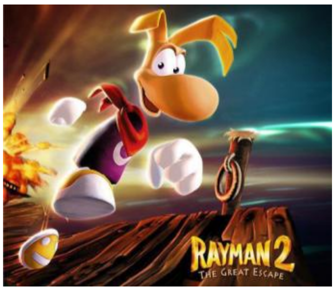

Links

beelden uit de “Dojo” video game

Rechts

beelden uit de “Rayman 2: The Great

Vertrouwelijkheid
We willen graag benadrukken dat alle gegevens vertrouwelijk zullen worden behandeld. Dat betekent dat alleen de onderzoekers van de universiteit jouw vragenlijst kunnen zien. Ook vul je bij de vragenlijst alleen een persoonlijke code in, jouw naam zal dus niet op de vragenlijst staan. Bovendien worden de gegevens uit de vragenlijsten nooit aan anderen gegeven. Je bent ook niet verplicht om deel te nemen aan het onderzoek.

Verdere informatie
Deze brief is bedoeld om jou wat meer informatie te geven over de rest van het onderzoek. We hopen dat jij en je ouders mee willen doen aan het onderzoek, omdat we er van overtuigd zijn dat jongeren heel veel aan deze weerbaarheidtrainingen kunnen hebben. Zowel nu als in de toekomst. Daarnaast hebben we jou en jouw mening over de weerbaarheidtrainingen echt nodig omdat we heel graag willen weten of video games gebruikt kunnen worden als weerbaarheidtraining.

Als je vragen hebt over het onderzoek, dan mag je altijd contact opnemen met Hanneke Scholten (024-3612551). Je kunt ook een e-mail sturen naar h.scholten@pwo.ru.nl.

Bedankt voor het lezen van deze brief!

Vriendelijke groeten namens het projectteam,

Hanneke Scholten

Projectteam:
Prof. Dr. Isabela Granic (Radboud Universiteit Nijmegen)
Prof. Dr. Rutger Engels (Radboud Universiteit Nijmegen)
Adam Lobel, Phd student (Radboud Universiteit Nijmegen)
Hanneke Scholten Bsc. (Radboud Universiteit Nijmegen)

Onderzoek naar de effectiviteit van computerspellen als weerbaarheidtraining

Hierbij stem ik in met deelname aan het onderzoek. Dit houdt in dat mijn kind op drie verschillende momenten vragenlijsten invult en gedurende drie weken twee keer per week meedoet aan de weerbaarheidtraining. Ik bevestig genoeg informatie te hebben ontvangen en goed over deelname aan het onderzoek heb kunnen nadenken. Ik begrijp dat deelname geheel vrijwillig en vrijblijvend is en dat ik op ieder moment mag stoppen zonder daarvoor een geldige reden op te hoeven geven. Ik geef toestemming voor het gebruik van de data (geanonimiseerd) die bij dit onderzoek verkregen wordt.

Vul hieronder de gegevens van uw kind in

Achternaam en voornaam : ……………………………………………………………………………………………………

Geboortedatum : ……………………………………………………………………………………………………

Jongen / meisje :……………………………………………………………………………………………………

Klas : ……………………………………………………………………………………………………

School :

Vul hieronder uw eigen gegevens in

Achternaam en voorletters : ……………………………………………………………………………………………………

Relatie tot uw kind (bijv. moeder) :……………………………………………………………………………………………………

Telefoon nummer : ……………………………………………………………………………………………………

Email adres : ……………………………………………………………………………………………………

Datum van vandaag : ……………………………………………………………………………………………………

Handtekening

…………………………………………………………
