# Supplementary material for: A Randomized Controlled Trial to Test the Effectiveness of an Immersive 3D Video Game for Anxiety Prevention among Adolescents
Source: PLoS One. 2016 Jan 27;11(1):e0147763. doi: 10.1371/journal.pone.0147763 (PMC4729475; doi:10.1371/journal.pone.0147763)
Supplement: S2 Protocol — (PDF) [file pone.0147763.s005.pdf]

APPLICATION ETHICAL COMMITTEE BEHAVIOURAL RESEARCH (ECG)

I. GENERAL GUIDELINES FOR THE USE OF THIS FORM

This form must be completed for each study and submitted to the ethical committee behavioural research (ECG) of the Faculty of Social Sciences of the Radboud University Nijmegen. Only if this form is completed the ECG will formulate a recommendation.

1. This form can be used for an individual experiment (project) or a line of research. Researchers are encouraged to submit research lines in which multiple experiments are proposed which are substantively linked and in which the same procedures and populations are used. Only lead investigators can submit a protocol, via their email address at the University of Nijmegen.
2. Ethical approval for a project or research line is valid for a period of five years or until a change occurs in the following disclosures. Researchers must annually inform the ECG if the submitted protocol is still active. When a project or research line is terminated, the ECG should be immediately informed.
3. The below mentioned researcher and collaborators have to treat participants according to the principles of the Declaration of Helsinki (see ECG website), and have to ensure that participants (mentally competent or incompetent and in a dependent relationship to the investigator or not) may at any time withdraw from participation without any consequences.
4. The researcher has to maximize the quality of research, statistical analysis and reporting, and has to respect specific regulation in relation to specific methods (e.g. FMRI).
5. The researcher declares that the research has been described truthfully, with special emphasis on ethical aspects.

For approval

Professor	Prof. dr. I. Granic
Director Research Institute	Prof. dr. A.H.N. Cillessen
Date	24Sep2013

II. GENERAL INFORMATION

Title	A randomized controlled trial to test the effectiveness of an immersive 3D video game in preventing anxiety.
Subsidieverstrekker	BSI
Name project supervisor	Isabela Granic
E-mail address project supervisor	i.granic@pwo.ru.nl
Name project executor	Hanneke Scholten
E-mail address project	h.scholten@student.ru.nl

executor

Research location High schools

Summarize the research project (maximum of 500 words, with emphasis on description of procedures, methods, and participants). If applicable, please provide sufficient information on the use of manipulations or stimulus materials.

Summary
One of the most popular leisure activities among youth in the United States is playing video games, with 97% of youth between ages 12-17 playing them (Lenhart, Kahne, Middaugh, Macgill, Evans, & Vitak, 2008). In the last five years more research has focused on potential cognitive, social, motivational and emotional benefits for youth provided by video games (Olson, 2010). Consequently, video games could learn youth new forms of strategies, behavior and thought and thereby could be used in a mental health context (Granic, Lobel & Engels, under review). Anxiety disorders are found to be one of the most common adolescent psychiatric disorders and have several negative consequences in different domains, such as interpersonal, emotional or academic functioning (Costello, Egger, Copeland, Erkanli, & Angold, 2011; Pine, 1997). Moreover, anxiety during adolescence is found to persist into adulthood and is related to other serious conditions, such as substance abuse or depression (Cartwright-Hatton, Roberts, Chitsabesan, Fothergill, & Harrington, 2004; Woodward & Fergusson, 2001).The present study will tap into the new area of video game research by testing a video game designed to prevent anxiety in adolescents.

Approximately thousand adolescents between 12 and 14 years of age will be recruited through schools in the region of Nijmegen and will be screened using the Spence Children's Anxiety Scale (SCAS), with passive consent from their parents. Adolescents showing elevated levels of anxiety are asked to participate in the study with active consent from themselves and their parents. Adolescents already receiving mental health care will be excluded from the study.

After screening, 120 participants will be randomly assigned to the experimental or control condition (with equal gender distribution). The experimental group will play the anxiety-reducing video game and the control group a different game for at least 6-8 hours spread over 6 weeks. The experimental game is called "Dojo" and is a challenging, motivating and entertaining video game using emotion regulation strategies (muscle relaxation, breathing techniques, positive self-talk) and HRV biofeedback to reduce anxiety in adolescents. HRV biofeedback will be measured by the Lightstone IOM device, which is non-invasive since the three sensors are attached to three fingertips of the participant. The control game (Super Mario World) is as engaging and difficult as the experimental game, but does not incorporate any anxiety reducing techniques. Participants will fill out the SCAS, Skills and Difficulties Questionnaire and Coping Questionnaire Child before the six week period of video game play, after this period and as a 3-month follow-up. Participants will receive €40,- as a compensation for filling in questionnaires after the 3-month follow-up. The confidentiality of these questionnaires is assured through the use of personal codes, which allow questionnaire data to be stored separate from personal data.

It is hypothesized that, compared to the control group, participants in the experimental condition will show significantly reduced anxiety. Furthermore, we expect that participants in the experimental condition who demonstrate a better understanding of their state of physical arousal will show a larger reduction in anxiety than participants in the experimental condition without or with little understanding of their state of physical arousal.

References
Cartwright-Hatton, S. (2006). Anxiety of childhood and adolescence: Challenges and opportunities. Clinical Psychology Review, 26, 813–816.

doi:10.1016/j.cpr.2005.12.001

Cartwright-Hatton, S., Roberts, C., Chitsabesan, P., Fothergill, C., & Harrington, R. (2004). Systematic review of the efficacy of cognitive behavioural therapies for childhood and adolescent disorders. British Journal of Clinical Psychology, 43, 421-436. doi: 10.1348/0144665042388928

Costello, E. J., Egger, H. L., Copeland, W., Erkanli, A., & Angold, A. (2011). The developmental epidemiology of anxiety disorders: phenomenology, prevalence, and comorbidity. In W.K. Silverman & A.P. Field (Eds.), Anxiety disorders in children and adolescents (pp. 56-75). New York: Cambridge University Press.

Granic, I., Lobel, A., & Engels, C. M. E. (n.d.). The benefits of playing video games. Unpublished manuscript.

Lenhart, A., Kahne, J., Middaugh, E., Macgill, A. R., Evans, C., & Vitak, J. (2008). Teens, video games, and civics. Pew Internet & American Life Project. Retrieved from: http://www.pewinternet.org/Reports/2008/Teens-Video-Games-and-Civics.aspx

Olson, C. K. (2010). Children's motivation for video game play in the context of normal development. Review of General Psychology, 14, 180-187. doi: 10.1037/a0018984

Pine, D. S. (1997). Childhood anxiety disorders. Current Opinion in Pediatrics, 9, 329-339.

Woodward, L. J., & Fergusson, D. M. (2001). Life Course Outcomes of Young People With Anxiety Disorders in Adolescence. Journal of the American Academy of Child & Adolescent Psychiatry, 40, 1086-1093. doi: 10.1097/00004583-200109000-00018

Do you have submitted this or similar studies to the ECG before?

(x) No
( ) Yes, ECG-number

Does this study concern a research project or a research line?

Research project

III. ETHICAL GUIDELINES

1. Here are some regulations that must be met according to the ECG. The observance of these regulations is the responsibility of the individual researcher or his/her supervisor. Are regulations below (A-D) met?

A. Research is expected to provide new and important insights.
B. Research meets requirements of correct methodology.
C. The study is performed under supervision of a competent person. The person carrying out possible interventions must be knowledgeable and experienced.
D. Research is not carried out with participants that are, outside the research project, in a subordinate position with respect to the investigator (e.g. own children).

(x) Yes
( ) No, explanation:

2. Is there a contact person who participants can ask questions about the research and will they be informed about this?

(x) Yes
( ) No, explanation:

3. Is it clear where complaints about study participation can be expressed and how these complaints will be treated?

(x) Yes
( ) No, explanation:

4. Are participants completely free to participate in this study, and may they withdraw from participation at any time without any consequences for whatever reason?

(x) Yes
( ) No, explanation:

5. Are participants and/or their representatives before participation informed about the purpose, nature and duration, risks and drawbacks of the study, and do they sign for participation by the standard informed consent (see the forms at the website)?

It is standard that participants or their legal representative

a. are fully informed (orally and written) in advance about the nature of the research

b. give written consent with the appropriate Consent Form

c. Feedback: are notified (orally and written) afterwards of the purpose of the investigation

The information is clear and appropriate to the participant (e.g. in case of minors). The subject receives reflection between information and consent. If full disclosure of information is impossible before the start of the study because of the nature of the investigation, the subject must be informed afterwards. Also, minors and incapacitated subjects should still be informed to comprehension in all cases.

( ) Yes
(x) No, explanation:
Schools wishing to participate in the study receive, before screening, letters in which all participants and their parents/guardians are informed about the study. In these letters, permission is sought from the parents / guardians for their child's participation in the screening through passive consent. It is indicated to parents that children will be

screened on a number of factors to take part in the study and that their child may be invited to participate in the continuation of the study. Parents have the possibility to refuse participation of their child through passive consent. Parents do not need to explicitly consent for the screening. This has several reasons:

a) Previous research on the effects of depression prevention programs (ECG2012-2711-069: Poppelaars/Lichtwarck; A randomized controlled trial comparing school-based (Op Volle Kracht) and computerized (SPARX) depression prevention programs with adolescent girls) showed that parents were satisfied with the application of passive consent. Moreover, schools have also expressed their preference for passive consent because parents still have the ability to give no consent for their child to participate.

b) It prevents delays in research and high non-response from parents / guardians.

Participants who are selected for the continuation of the study will be called, informed about the results of the screening and asked to give permission or refuse to participate in the continuation of the investigation. Also, parents have to give explicit consent at this point in the study for participation of their child in the continuation of the study (active consent).

6. In case there is deception, does the procedure then comply with the standard requirements (no deception about the risks, accurate debriefing)?

Deception refers to the provision of inaccurate or incomplete information to the participant. Deception is only permitted if there is no possibility to answer the question without deception. Deception is not allowed when it comes to information about the possible risks associated with participation. After deception, there is always a full debriefing for the participant how he or she was misguided. If there is are temporary negative effects expected of deception, then debriefing should be done immediately after the end of the experiment (e.g., if false negative feedback was given on intelligence scores, there will be an immediate debriefing). The debriefing is held in a way that can be expected that temporary negative effects on for example self-esteem or mood are removed by debriefing. When no temporary negative effects are expected, the debriefing may also take place at a later time, but no later than one month after the end of the experiment.

(x) There is no deception
( ) There is deception that complies with the standard requirements
( ) There is deception that doesn't comply with the standard requirements (explanation)

7. Is there compliance with the standard rules relating to anonymity and privacy?

The research data is standard

a. processed anonymously and stored confidentially

b. including access rights for the participant of personal data

c. with access to all data for all researchers involved in the project

(x) Yes
( ) No, explanation:

IV. PARTICIPANTS

8. Is it a healthy population?

(x) Yes
( ) No, explanation:

9. Are participants screened to reduce the risk of adverse effects of the study?

(x) No
( ) Yes, explanation:

10a. Is there research on minors (<18 years old) or incapacitated?

Incapacitated are people who are unable to make a reasonable assessment of their interests in the matter.

(x) Yes, go to question 10b
( ) No, go to question 11

10b. Is it research for a specific target population?

Research for a specific population is research that cannot be performed without participation of people belonging to this population (e.g., minors, demented elderly).

(x) Yes
( ) No, explanation:

10c. Is there a practical reason why minors or incapacitated participate in the research, for example because not enough mentally competent people can be recruited?

(x) No
( ) Yes, explanation:

10d. Is it possible to first obtain the necessary knowledge with adult, mentally competent people?

(x) No
( ) Yes, explanation:

10e. Is this therapeutic research, which means that the study can also be beneficial to the participant itself?

(x) Yes
( ) No, explanation:

V. THE METHOD USED

11. Are there methods used that could possibly identify incidental findings of which the participant should be informed?

Some methods of investigation may provide incidental findings that may be relevant to the subject. These include cardiac arrythmia on an ECG, abnormal EEG (epilepsy), or a deviation on a fMRI. If such a possibility exist, a clause should be included in the informed consent that provides the procedure to be followed then. The participant should specify the name and place of practice of his / her GP, who will be notified in the event of relevant findings. If the participant does not have a GP than he/she should agree with the fact that the student doctor, or a company doctor is notified in such cases. The subject must agree with this procedure by signing a separate clause on the informed consent form.

( ) No
(x) Yes, explanation:
While playing the video game Dojo participants will be connected to an IOM HRV biofeedback system. However, this system does not give a complete overview of the ECG of the participant. It gives a number in a certain range that reflect beats per minute (BPM). Since this output gives too little information about the ECG of the participant, there is no possibility that cardiac arrhythmia or other heart conditions may come forward.

12. Are unobtrusive methods used?

When unobtrusive methods are used, data is collected without the participant knowing this. The behavior of the subject is for example observed or videotaped without them knowing.

(x) No
( ) Yes, explanation:

13. Are participants subjected to acts or imposed to a particular mode of behavior that fall outside the normal way of life?

Examples: stress generation, exert pressure to exceed significant values, induce false memories, processing of serious events, prolonged or very frequent interrogation,

ambulatory measurements, effectiveness of interventions, evoking unpleasant psychological or physical symptoms within a experiment

( ) No
(x) Yes, explanation:
In this study the effectiveness of two video games will be tested in the prevention of anxiety. These video games are primarily focused on everyday stressors and fearful situations for adolescents. There will be no deliberate generation of stress, pressure to exceed important values, inducing false memories, treatment of serious events, prolonged or very frequent interrogation, ambulatory measurements, effectiveness of interventions. However, it is possible that the video game will evoke anxiety in the adolescent, accompanied by unpleasant psychological or physical symptoms. Though, the video game offers the tools to overcome this fear and unpleasant symptoms. The trainer will closely monitor the participants and any signs of intense anxiety will be discussed with the project executor.

14. Are the risks and drawbacks minimal? Are the risks as high as in normal life?

(x) Yes
( ) No, explanation:

15. Is there another compensation offered to the participants besides the standard compensation?

(x) No
( ) Yes, explanation:

VII. ADDITIONAL FORMS

Along with this form, the following documents must be submitted:

- The text of the ads or letters;
- The text of the written feedback (debriefing) indicating contact information of responsible researcher;
- A copy of the consent form;

Radboud Universiteit Nijmegen

Faculteit der Sociale Wetenschappen

Radboud Universiteit Nijmegen

T.a.v. Hanneke Scholten

Orthopedagogiek Gezin & Gedrag, A.06.11a

Montessorilaan 3

6525 HR Nijmegen

Tel: 024-3612551

Fax: 024-3612776

Email: h.scholten@pwo.ru.nl

www.ru.nl/fsw

Telefoonnummer	Datum
024-3612551	23 oktober 2013

Betreft	E-mail
Uitnodiging deelname weerbaarheidstudie	h.scholten@pwo.ru.nl

Beste meneer/mevrouw,

Zoals u op de flyer kunt lezen zijn wij bezig met een onderzoek naar twee video games die worden geëvalueerd op hun effectiviteit. In dit onderzoek richten wij ons specifiek op het weerbaar maken van jongens en meisjes tijdens hun adolescentie. We zullen onderzoeken welke van de twee video games effectiever is in het aanleren van bepaalde strategieën aan jongeren, zoals doorzettingsvermogen, regulatie van emoties zoals angst, boosheid of frustratie en het omgaan met tegenslagen en falen. Wij sturen u deze flyer en brief met het verzoek om aan ons onderzoek deel te nemen.

Op scholen die deelnemen aan het onderzoek zullen wij in november of december 2013 een vragenlijst afnemen bij alle jongens en meisjes van het eerste en tweede jaar. Deze vragenlijst gaat o.a. over hoe de jongeren denken, wat ze voelen en hoe ze met problemen omgaan. Vervolgens zullen de jongeren worden geselecteerd om deel te nemen aan het onderzoek.

Beide video games zullen door de jongeren die geselecteerd zijn op school maar na schooltijd of in tussenuren worden gespeeld. De jongeren zullen 6 uur verspreid over 3 weken (in de periode tussen januari en april 2014) één van beide video games spelen. Voorafgaand aan en na afloop van de 3 weken vullen de jongeren een aantal vragenlijsten in gericht op moeilijkheden die ze tegenkomen in het dagelijks leven en de manieren waarop ze daar mee om weten te gaan. Vervolgens wordt er 3 maanden na afloop van de 3 weken na schooltijd of in tussenuren een vragenlijst afgenomen om de veranderingen en effecten op de langere termijn te kunnen bepalen. Iedere deelnemende jongere ontvangt een vergoeding van €40,- als tegenprestatie voor het invullen van de vragenlijsten.

Wij hopen van harte dat u wilt deelnemen aan het onderzoek aangezien het een belangrijke bijdrage kan leveren aan de bevordering van de mentale gezondheid van Nederlandse jongeren. Als dank voor uw deelname bieden we na afloop van het onderzoek een rapport aan over de uitslag van het onderzoek. Ook bestaat de mogelijkheid deskundigheidsbevordering voor docenten te organiseren.

Wij realiseren ons dat het begin van het schooljaar druk is en houden deze brief dan ook bewust kort. Graag horen wij van u of u mee wil werken aan de weerbaarheidstudie. Wij bellen u hierover binnen een week terug. U kunt hiervoor ook zelf contact opnemen via de mail (h.scholten@pwo.ru.nl), of bellen naar 024-3612551. Natuurlijk kunt u hier ook terecht voor vragen of meer informatie.

Namens het hele projectteam dank ik u hartelijk voor uw aandacht en een eventuele reactie,

Hanneke Scholten

Projectteam:

Prof. Dr. Isabela Granic (Radboud Universiteit Nijmegen)

Prof. Dr. Rutger Engels (Radboud Universiteit Nijmegen)

Adam Lobel, Phd student (Radboud Universiteit Nijmegen)

Hanneke Scholten Bsc. (Radboud Universiteit Nijmegen)

Radboud Universiteit Nijmegen

Onderzoek naar effectiviteit weerbaarheidtrainingen op middelbare scholen

Geachte ouder(s)/verzorger(s),

Met deze brief willen wij u graag informeren over het verdere verloop van het onderzoek naar de effectiviteit van twee weerbaarheidtrainingen voor jongeren met verminderde weerbaarheid en assertiviteit.

Zoals u weet vindt het Over Betuwe College Huissen dit onderzoek een meerwaarde voor de zorg voor haar leerlingen en heeft ingestemd met het uitvoeren hiervan. Ook de ethische commissie van de Faculteit der Sociale Wetenschappen van de Radboud Universiteit heeft haar goedkeuring gegeven voor de uitvoering van dit onderzoek. U heeft eerder al informatie ontvangen over het onderzoek en heeft toegestemd met deelname van uw kind aan de screening van het onderzoek. Met deze brief willen wij u informeren over de uitkomsten van deze screening en het verdere verloop van het onderzoek.

Waarom dit onderzoek?

Onzekerheid, verminderde mentale weerbaarheid en verminderde assertiviteit zijn problemen waar behoorlijk wat jongeren mee te maken hebben en die van grote invloed kunnen zijn op hun functioneren. Er is steeds meer bewijs dat video games een positieve invloed kunnen hebben op sociale, cognitieve en emotionele vaardigheden van jongeren. Daarnaast hebben video games als grote voordeel dat ze jongeren kunnen motiveren. Deze voordelen van video games hebben de potentie om jongeren weerbaarheidstrategieën aan te leren in een uitdagende context. Omdat het inzetten van video games in het weerbaar maken van jongeren een zeer nieuwe aanpak is, hebben wij uw zoon of dochter en u nodig om deze video games te evalueren. Daarnaast willen we met dit onderzoek bekijken hoe effectief beide trainingen zijn in het helpen van jongeren met milde klachten aangaande weerbaarheid, assertiviteit en onzekerheid.

Resultaat van de screening

Zoals u weet heeft uw kind deelgenomen aan de screening van het onderzoek. De screening hield in dat alle jongeren uit de tweede klas een vragenlijst hebben ingevuld. Met behulp van deze vragenlijst wilden we bekijken in welke mate uw kind klachten ervaart op het gebied van onzekerheid, assertiviteit en weerbaarheid. Zoals aan de telefoon ook al is besproken, bleek uit deze vragenlijst dat uw kind meer dan leeftijdsgenoten aangeeft problemen te ervaren op deze gebieden.

Wat houden de weerbaarheidtrainingen in?

Dit onderzoek richt zich op twee verschillende weerbaarheidtrainingen in de vorm van video games, “Dojo” en “Rayman 2: The Great Escape”. Beide video games zijn erop gericht jongeren bepaalde

strategieën aan te leren die ze kunnen helpen om meer weerbaar te worden. Vaardigheden als doorzettingsvermogen, omgaan met tegenslagen of falen en omgaan met emoties zullen aan bod komen in beide weerbaarheidtrainingen.

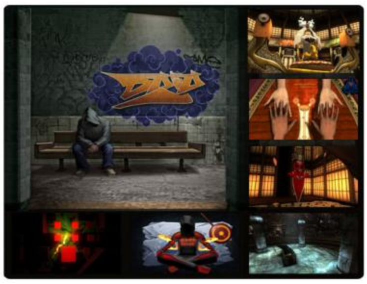

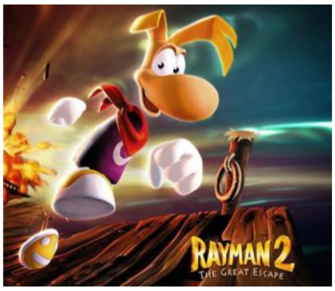

Links

beelden uit de “Dojo” video game

Rechts

beelden uit de “Rayman 2: The Great

Wij willen benadrukken dat beide video games geen therapeutische programma’s zijn, en zeker geen zwaarmoedige programma’s die zich richten op zware problemen. We geloven dat juist de inzet van luchtige, leuke en motiverende video games effectiever zal zijn in het weerbaar maken van jongeren.

Het verdere onderzoek

Omdat uw kind aangeeft meer dan gemiddeld klachten te ervaren van onzekerheid, assertiviteit en weerbaarheid, komt uw kind in aanmerking om deel te nemen aan het verdere onderzoek. Dit houdt in dat uw kind kan deelnemen aan één van de twee weerbaarheidtrainingen. Jongeren die op dit moment al in behandeling zijn voor dit soort klachten, kunnen helaas niet deelnemen aan het verdere onderzoek. De weerbaarheidtrainingen bestaan beide uit 6 sessies van een uur verspreid over drie weken. Deze sessies zullen op school, maar na schooltijd plaatsvinden. De weerbaarheidtrainingen zijn kosteloos.

Alle deelnemende jongeren wordt gevraagd om drie keer een vragenlijst in te vullen over hun angsten, zorgen en hun vaardigheden om daarmee om te gaan: in februari 2014, maart 2014 en juni 2014. Deze vragenlijsten ontvangt uw kind per mail en het invullen van de vragenlijsten zal per keer ongeveer 30 minuten in beslag nemen. Indien uw kind op alle drie de tijdsmomenten alle vragenlijsten heeft ingevuld en deelgenomen heeft aan alle zes trainingssessies (met uitzondering van afmelding met een goede reden zoals ziekte) ontvangt uw kind een vergoeding van 40,- euro.

De deelnemende jongeren worden willekeurig verdeeld over twee groepen. De ene helft van de jongeren zal het spel “Dojo” gaan spelen en de andere helft zal het spel “Rayman 2: The Great Escape” gaan spelen. Omdat de verdeling willekeurig is, hebben wij geen invloed op welke jongeren in welke groep worden geplaatst.

Vertrouwelijkheid

We willen graag benadrukken dat *alle* gegevens vertrouwelijk zullen worden behandeld en dat in geen enkel rapport namen vermeld zullen worden. Uitsluitend de onderzoekers van de universiteit hebben inzage in de vragenlijst. Bovendien worden de gegevens **nooit** aan derden verstrekt. Uiteraard zijn u en uw kind niet verplicht om deel te nemen aan het onderzoek.

Hoe verder?

Als bijlage bij deze brief vindt u de toestemmingsverklaring en een retourenvelop. Indien u toestemt met deelname van uw kind aan het verdere onderzoek, dient u deze toestemmingsverklaring in te vullen en te ondertekenen en uiterlijk 7 februari 2014 terug te sturen in de bijgevoegde retourenvelop. Nogmaals, u en uw kind zijn niet verplicht om deel te nemen. U kunt op elk moment stoppen met het onderzoek.

Wij hopen u met deze brief voldoende te hebben geïnformeerd. Wanneer u nog inhoudelijke vragen heeft over het onderzoek, aarzelt u dan niet om contact op te nemen met Hanneke Scholten (024-3612551). U kunt ook een e-mail sturen naar h.scholten@pwo.ru.nl.

Dank voor uw aandacht!

Vriendelijke groeten namens het projectteam,

Hanneke Scholten

Projectteam:

Prof. Dr. Isabela Granic (Radboud Universiteit Nijmegen)

Prof. Dr. Rutger Engels (Radboud Universiteit Nijmegen)

Adam Lobel, Phd student (Radboud Universiteit Nijmegen)

Hanneke Scholten Bsc. (Radboud Universiteit Nijmegen)

Radboud Universiteit Nijmegen

Omgaan met lastige situaties, hoe doe je dat? Een onderzoek op jouw school.

Beste leerling,

Op jouw school is een tijdje geleden een vragenlijst afgenomen bij alle leerlingen van de tweede klas. Daarbij is toen verteld dat een aantal leerlingen opgebeld zouden worden met de vraag of ze mee willen doen aan de rest van het onderzoek. Met deze brief willen we je graag wat vertellen over het onderzoek waarvoor wij deze vragenlijst hebben afgenomen en hoe het vervolg van het onderzoek er uit gaat zien.

Waarom dit onderzoek?
Als je in de leeftijd van 12 tot 14 jaar bent, kom je veel uitdagingen tegen. Je gaat bijvoorbeeld van de basisschool naar de middelbare school, je leert allemaal nieuwe mensen kennen, je probeert nieuwe vrienden te maken en je probeert goede cijfers te halen. Omdat er in deze periode zoveel dingen gebeuren, is het niet raar dat sommige jongeren dit af en toe lastig vinden. Sommige jongeren zijn bijvoorbeeld ontevreden over hun uiterlijk, anderen voelen zich soms alleen en een aantal jongeren is bang voor verschillende dingen. Omdat deze problemen best vaak voorkomen in de leeftijd van 12 tot 14 jaar worden er zogenaamde weerbaarheidtrainingen ontwikkeld die jongeren kunnen helpen om beter in hun vel te komen zitten. Weerbaarheidtrainingen leren jongeren om minder onzeker te zijn, voor zichzelf op te komen en zich beter te voelen. Wij hebben ervoor gekozen om twee video games te gebruiken als weerbaarheidtrainingen, namelijk "Dojo" en "Rayman 2: The Great Escape". Omdat video games nog nooit gebruikt zijn om jongeren weerbaar te maken, hebben we jou nodig om te testen of video games daarvoor geschikt zijn. Wat jij van de video game vindt is dus heel belangrijk!

De uitkomst van de vragenlijst
Wij willen, als onderzoekers van de Radboud Universiteit, graag onderzoeken of video games ervoor kunnen zorgen dat jij weerbaarder wordt. Om dit te kunnen onderzoeken, hebben we eerst een vragenlijst afgenomen bij alle leerlingen van het derde jaar. Deze vragenlijst ging over hoe je denkt, wat je voelt en hoe je met moeilijkheden omgaat. Zoals we je aan de telefoon ook al hebben verteld, bleek uit de vragenlijst dat jij je soms wat meer zorgen maakt of wat banger bent voor sommige dingen dan je leeftijdsgenoten.

Wat houden de weerbaarheidtrainingen in?
Dit onderzoek richt zich op twee verschillende video games, "Dojo" en "Rayman 2: The Great Escape". Beide video games leren je om door te zetten in een lastige situatie, om te gaan met tegenslagen en om te gaan met verschillende emoties zoals angst of boosheid.

Hoe ziet het onderzoek er precies uit?
Als je mee wilt doen aan het onderzoek, zul je drie weken lang twee keer per week één van de twee video games spelen. Dit zijn in totaal zes sessies die allemaal een uur duren. Voordat je gaat beginnen met het spelen van de video game, na afloop van de zes sessies en drie maanden na afloop

van de zes sessies zullen we jou vragen om een paar vragenlijsten in te vullen. Met de uitkomsten van die vragenlijsten kunnen wij testen of “Dojo” en/of “Rayman 2: The Great Escape” jongeren kunnen helpen om meer weerbaar te worden. Je wordt willekeurig ingedeeld in één van de twee groepen. De eerste groep zal het spel “Dojo” gaan spelen en de tweede groep zal het spel “Rayman 2: The Great Escape” gaan spelen. Omdat de verdeling willekeurig is, weten we niet in welke groep je terecht zal komen. Als je meedoet aan alle zes sessies en op alle drie de tijdsstippen alle vragenlijsten hebt ingevuld zul je een beloning van 40 euro ontvangen.

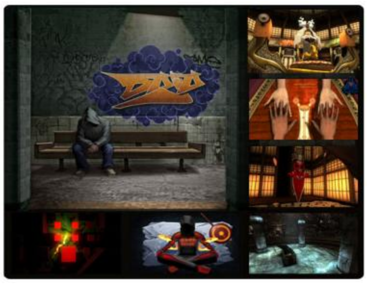

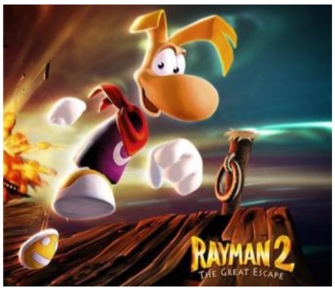

Links

beelden uit de “Dojo” video game

Rechts

beelden uit de “Rayman 2: The Great

Vertrouwelijkheid
We willen graag benadrukken dat alle gegevens vertrouwelijk zullen worden behandeld. Dat betekent dat alleen de onderzoekers van de universiteit jouw vragenlijst kunnen zien. Ook vul je bij de vragenlijst alleen een persoonlijke code in, jouw naam zal dus niet op de vragenlijst staan. Bovendien worden de gegevens uit de vragenlijsten nooit aan anderen gegeven. Je bent ook niet verplicht om deel te nemen aan het onderzoek.

Verdere informatie
Deze brief is bedoeld om jou wat meer informatie te geven over de rest van het onderzoek. We hopen dat jij en je ouders mee willen doen aan het onderzoek, omdat we er van overtuigd zijn dat jongeren heel veel aan deze weerbaarheidtrainingen kunnen hebben. Zowel nu als in de toekomst. Daarnaast hebben we jou en jouw mening over de weerbaarheidtrainingen echt nodig omdat we heel graag willen weten of video games gebruikt kunnen worden als weerbaarheidtraining.

Als je vragen hebt over het onderzoek, dan mag je altijd contact opnemen met Hanneke Scholten (024-3612551). Je kunt ook een e-mail sturen naar h.scholten@pwo.ru.nl.

Bedankt voor het lezen van deze brief!

Vriendelijke groeten namens het projectteam,

Hanneke Scholten

Projectteam:
Prof. Dr. Isabela Granic (Radboud Universiteit Nijmegen)
Prof. Dr. Rutger Engels (Radboud Universiteit Nijmegen)
Adam Lobel, Phd student (Radboud Universiteit Nijmegen)
Hanneke Scholten Bsc. (Radboud Universiteit Nijmegen)

Onderzoek naar de effectiviteit van computerspellen als weerbaarheidtraining

Hierbij stem ik in met deelname aan het onderzoek. Dit houdt in dat mijn kind op drie verschillende momenten vragenlijsten invult en gedurende drie weken twee keer per week meedoet aan de weerbaarheidtraining. Ik bevestig genoeg informatie te hebben ontvangen en goed over deelname aan het onderzoek heb kunnen nadenken. Ik begrijp dat deelname geheel vrijwillig en vrijblijvend is en dat ik op ieder moment mag stoppen zonder daarvoor een geldige reden op te hoeven geven. Ik geef toestemming voor het gebruik van de data (geanonimiseerd) die bij dit onderzoek verkregen wordt.

Vul hieronder de gegevens van uw kind in

Achternaam en voornaam : ……………………………………………………………………………………………………

Geboortedatum : ……………………………………………………………………………………………………

Jongen / meisje :……………………………………………………………………………………………………

Klas : ……………………………………………………………………………………………………

School :

Vul hieronder uw eigen gegevens in

Achternaam en voorletters : ……………………………………………………………………………………………………

Relatie tot uw kind (bijv. moeder) :……………………………………………………………………………………………………

Telefoon nummer : ……………………………………………………………………………………………………

Email adres : ……………………………………………………………………………………………………

Datum van vandaag : ……………………………………………………………………………………………………

Handtekening

…………………………………………………………
